# Asymmetrically Disubstituted Pyrenebutyrate Complexes of Pt(IV) as Cisplatin Prodrugs with Improved Anticancer Activity

**DOI:** 10.3390/molecules31132336

**Published:** 2026-07-03

**Authors:** Rositsa Mihaylova, Veronika Mihaylova, Nikola Burdzhiev, Ivo D. Ivanov, Zhanina Petkova, Georgi Momekov, Denitsa Momekova, Anife Ahmedova

**Affiliations:** 1 Faculty of Pharmacy, Medical University—Sofia, 2 Dunav Street, 1000 Sofia, Bulgaria; rmihaylova@pharmfac.mu-sofia.bg (R.M.); dmomekova@pharmfac.mu-sofia.bg (D.M.); 2 Faculty of Chemistry and Pharmacy, Sofia University, 1, J. Bourchier blvd., 1164 Sofia, Bulgaria; 3 Institute of Organic Chemistry with Centre of Phytochemistry, Bulgarian Academy of Sciences, Acad. G. Bonchev Str. Bl. 9, 1113 Sofia, Bulgaria; zhanina.petkova@orgchm.bas.bg

**Keywords:** platinum(IV) prodrugs, anticancer agents, cytotoxicity, cellular uptake, activation by reduction, proteome analysis, apoptosis

## Abstract

Among the non-classical platinum complexes, Pt(IV) prodrugs are most promising as versatile scaffolds for structural modification and fine tuning of their activation-by-reduction mechanism of action and the resulting anticancer activity. Herein, four new asymmetrically disubstituted pyrenebutyrate complexes of Pt(IV) (**2**–**5**) were synthesized and thoroughly studied. In this series, the second axial ligand was derived from dicarboxylic acids of different length—4 and 5 C-atoms, or replacement of the C-atom in the middle with either O- or S-atom. The structural effects on reduction kinetics, lipophilicity and cellular internalization of the complexes were monitored by NMR, HPLC, fluorescence and ICP-MS measurements. Their cytotoxicity was tested on a panel of cancer cell lines and mechanistic insights were obtained from proteome analysis and microscope imaging. The data indicate that all complexes, especially complex **3**, represent a promising class of Pt(IV) prodrugs, exhibiting significantly higher cytotoxic activity than cisplatin in all tested models, including a cisplatin-resistant line. This was explained with a stronger and more integrated apoptotic response than cisplatin: pronounced Bax upregulation (3.6-fold), maximal cleaved caspase-3 (4-fold), activation of both intrinsic and extrinsic pathways, and effective p53 Ser15/Ser46 phosphorylation. The consistent rank order of potency (**3** > **4** > **5** ≈ **2** ≫ cisplatin) suggests that subtle ligand modifications can substantially enhance efficacy, possibly by improving cellular uptake or altering DNA binding.

## 1. Introduction

The design of Pt(IV) prodrugs represents a sophisticated evolution in metal-based chemotherapy, aimed at overcoming the limitations and systemic toxicities associated with traditional agents like cisplatin [1,2,3]. The primary architectural advantage of Pt(IV) complexes lies in their octahedral geometry. This structure enables an “activation-by-reduction” mechanism [4,5,6], in which the complex remains relatively inert until it reaches the reducing intracellular environment of cancer cells. This controlled activation is the cornerstone for achieving a superior selectivity profile and reducing the severe off-target reactivity and side effects characteristic of FDA-approved Pt drugs [7].

Furthermore, Pt(IV) complexes offer several pharmacological advantages, including improved administration routes, structural tunability, high potency, and compatibility with advanced delivery systems. Remarkable achievements have been attained exploiting some of these advantages. The increased stability of the Pt(IV) complexes has enabled the development of orally bioavailable drugs, such as satraplatin, which showed significant therapeutic promise in clinical trials. Well-established synthetic protocols now allow researchers to precisely tune both axial and equatorial ligands. This flexibility enables the creation of multitargeted “dual-action” drugs by attaching biologically active axial ligands—such as HDAC inhibitors or NSAIDs [8,9,10,11,12]—to the platinum core. Upon reduction, these complexes release multiple bioactive components to work synergistically. Modern Pt(IV) designs have achieved remarkable cytotoxicity, often reaching the nanomolar IC_50_ range [13,14]. This potency is maintained or improved by incorporating diverse axial groups, including enzyme inhibitors [12,15], long alkyl chains [16,17], or bulkier aromatic carboxylates [18,19]. Even endogenous molecules like melatonin have been successfully utilized to create highly potent prodrugs [20]. The enormous potential in the Pt designs is exploited in their adaptation for specialized delivery platforms.

Despite these advantages, several significant challenges remain in the design and clinical translation of these agents. Transitioning from promising lab results to clinical success remains difficult. Even highly anticipated candidates like satraplatin have faced setbacks, with some studies being abandoned at Phase III. This highlights the ongoing need to refine the therapeutic and reactivity profiles of new complexes [11,21,22,23,24,25,26]. A major conceptual challenge is whether a single multitargeted Pt complex can truly outperform established clinical regimens that use flexible, concomitant combinations of separate drugs [27]. Optimizing a prodrug requires a delicate balance between chemical reactivity and physical properties. Researchers must carefully correlate lipophilicity and ligand stability with cytotoxic performance [18,28,29,30].

**Figure 1:**
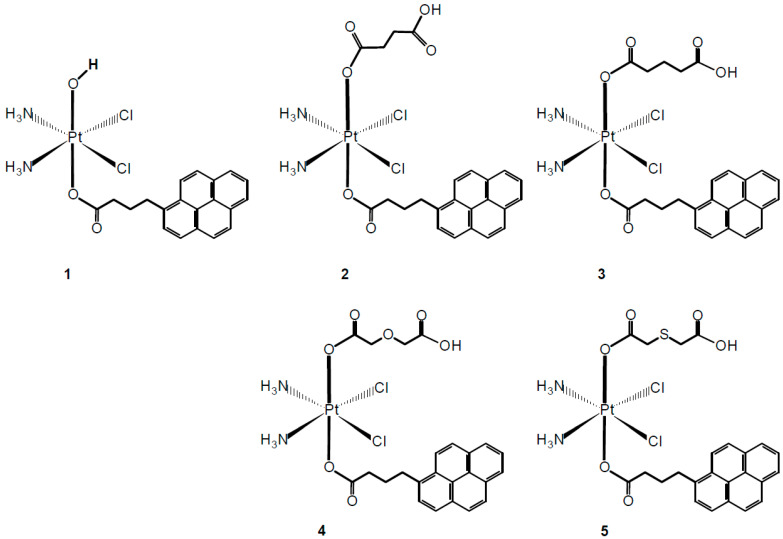
Structure of the studied asymmetrically disubstituted pyrenebutyrate Pt(IV) complexes (**2**–**5**) derived from the monosubstituted complex **1**.

In this context, the integration of Pt(IV) prodrugs with supramolecular delivery strategies has emerged as a promising direction to address these limitations. Building on this concept, we designed Pt(IV) complexes functionalized with a pyrenebutyric axial ligand, inspired by our previous studies on metallosupramolecular coordination capsules, where pyrene demonstrated strong host–guest interactions and selective modulation of anticancer activity [31,32]. Pyrene was selected as a functional moiety due to its strong π–π stacking interactions, which facilitate stable host–guest complex formation and enable selective incorporation into supramolecular delivery systems. Similar examples have been reported on arene–ruthenium supramolecular system that employ pyrenyl-modified dendrimers to improve drug delivery [33,34]. In this context, we envisioned Pt(IV) complexes as suitable guest molecules that could exploit such supramolecular platforms for targeted delivery. By functionalizing Pt(IV) complexes with such recognition elements, we aim to align with these guidelines and facilitate more effective delivery to tumor sites. However, efficient therapeutic action requires the release of the active Pt(II) species, which can be achieved via reductive cleavage of the axial ligands. While pyrene itself may remain trapped within the host system, its role as a recognition element is preserved, facilitating delivery without contributing directly to cytotoxicity, despite existing reports on the intrinsic toxicity of some pyrene derivatives [35]. Building on our earlier findings [14], which revealed significant differences between mono- and bis-substituted derivatives, we investigate here a series of asymmetrically disubstituted Pt(IV) complexes (**2**–**5** in Figure 1, derived from the monosubstituted pyrenebutyric complex **1**). Their cytotoxicity, cellular uptake, and reduction-dependent activation were evaluated to establish structure–activity relationships and assess their potential as supramolecularly delivered anticancer agents.

## 2. Materials and Methods

### 2.1. Apparatus and Materials

IR spectra were recorded in KBr pellets using a Nicolet 6700 Thermo Scientific FT-IR spectrometer (Thermo Fisher Scientific, Waltham, MA, USA). NMR spectra were obtained on a Bruker Avance III HD (Rheinstetten, Germany) (500.13 MHz for ^1^H and 107.5 MHz for ^195^Pt NMR). The chemical shifts are given in parts per million (δ) in DMSO-*d*_6_ relative to the residual solvent peak. The high-resolution mass spectra were recorded on a Q Exactive Plus Hybrid Quadrupole-Orbitrap Mass Spectrometer Thermo Scientific (Bremen, Germany) (HESI HRMS) in positive and negative mode. The spectra were processed using the Thermo Scientific FreeStyle program version 1.8 SP1 (Thermo Fisher Scientific Inc., Waltham, MA, USA). The reduction of the complexes was followed by testing on a high-performance liquid chromatography (HPLC) system equipped with an ODS (Macherey-Nagel Nucleodur, Dueren, Germany, 100-5, C-18, 150 × 4.6 mm and 5 µm particle size) chromatography column, including a quaternary pump “Prominence” UPLC LC-20AD, Shimadzu (Kyoto, Japan); degasser “Spectra System 1000”, Thermo Scientific (Waltham, MA, USA); UV/VIS detector “Spectra System UV 2000”, Thermo Scientific (Waltham, MA, USA); and software package “ClarityLite” (version 10.1, Chromatographic Station, Data Apex, Prague, Czech Republic). The determination of platinum content in cells was performed by ICP-MS analysis on a Perkin Elmer SCIEX DRC-e inductively coupled plasma mass spectrometer (MDS Inc., Concord, ON, Canada), equipped with a cross-flow nebulizer. The mass spectrometer was optimized to ensure maximum intensity of the Pt isotopes. The instrumental conditions for ICP-MS are described in [14]. Fluorescence spectra were recorded on a “Cary Eclipse” spectrophotometer (Varian, Mulgrave, Victoria, Australia). Fluorescence imaging of cancer cells was performed using an OPTIKA microscope (Ponteranica, Italy) equipped with an Optikam PRO6 digital camera. Thirty per cent hydrogen peroxide, potassium tetrachlorido platinate(II), N-hydroxysuccinimide, 1-pyrenebutyric acid and diglycolic anhydride were purchased from Sigma-Aldrich, Taufkirchen, Germany. The succinic, glutaric and thiodiglycolic anhydride were prepared by dehydration of the corresponding acids. All solvents were of AR or synthetic grade and were used without further purification. All in vitro experiments used cis-diaminodichlorido platinum(II) and cisplatin obtained from Sigma-Aldrich Chemie GmbH, Taufkirchen, Germany. All buffer solutions were freshly prepared with deionized water before use.

### 2.2. Synthesis and Characterization

Complex **1** was synthesized following the literature procedure for modification of the starting Pt(IV) analogue of cisplatin—oxoplatin, *c,c,t*-[Pt(NH_3_)_2_Cl_2_(OH)_2,_], as described by Erxleben and coworkers [9]. The series of asymmetrically disubstituted complexes **2**–**5** were obtained by reacting **1** (ca. 0.17–0.24 mmol) with 4-fold excess of the corresponding anhydride, namely succinic, glutaric, diglycolic and thiodiglycolic anhydrides, respectively. The reaction mixture was stirred at 60 °C for 24 h in DMF (6 mL). After reducing the DMF solvent to 2 mL by evaporation, the resulting complex was precipitated with diethyl ether (150 mL). The pale-yellow solids were isolated by centrifugation, washed twice with diethyl ether and dichloromethane, dried and stored under vacuum. IR, NMR (^1^H and ^195^Pt) and HRESI-MS spectra were used for structural characterization of the complexes and are provided in the Supplementary Materials, Figures S1–S18.

Complex **2**: (yield, 138 mg; 0.196 mmol; 82.5% relative to Pt). C_24_H_26_Cl_2_N_2_O_6_Pt, MW = 703.082: HESI-HRMS, calcd for C_24_H_25_Cl_2_N_2_O_6_Pt^−^ [M-H]^−^ = 701.07218 *m*/*z*, found 701.07385 *m*/*z*, Δ = 2.4 ppm; HESI-HRMS, calcd for C_24_H_27_Cl_2_N_2_O_6_Pt^+^ [M+H]^+^ = 703.08673 *m*/*z*, found 703.08557 *m*/*z*, Δ = −1.6 ppm; ^1^H NMR (DMSO-d_6_) 1.97 (qv, 2H, *J* = 7.6 Hz, CH_2_), 2.35–2.42 (m, 2H, CH_2_), 2.51 (t, 2H, *J* = 7.1 Hz, CH_2_), 3.36 (t, 2H, *J* = 7.7 Hz, CH_2_), 6.54 (br. s, 6H, NH_3_), 7.98 (d, 1H, J = 7.8 Hz, HPyr), 8.05 (t, 1H, J = 7.6 Hz, HPyr), 8.11 (d, 1H, J = 9.0 Hz, HPyr), 8.14 (d, 1H, J = 9.0 Hz, HPyr), 8.19–8.23 (m, 2H, HPyr), 8.26 (t, 2H, J = 8.0 Hz, HPyr), 8.46 (d, 1H, J = 9.3 Hz, HPyr), 12.06 (br. s, 1H, COOH); ^195^Pt NMR (DMSO-d_6_) δ: 1227 ppm; IR (ν, cm^−1^): 3560 (br, ν_O-H_), 3242 (s, ν_N-H_), 3233 (s, ν_N-H_), 3114 (s, ν_N-H_), 3044 (s, ν_N-H_), 2932 (m, ν_C-H_), 1645 (vs, ν_C=O_), 1615 (s, ν_C=C_), 1558 (m, ν_C-O_), 1434 (w, ν_C-C_), 1417 (m, ν_C-C_), 1381 m, 1320 s, 1287 m, 1254 m, 1193 (s, δ_C-H_), 1104 (w, δ_C-H_), 843 (s, γ_C-H_); 672 (m, γ_C-H_); br, s, m, w, vs stand for broad, strong, medium, weak and very strong, respectively.

Complex **3**: (yield, 85 mg; 0.119 mmol; 45.6% relative to Pt). C_25_H_28_Cl_2_N_2_O_6_Pt, MW = 717.097: HESI-HRMS, calcd for C_25_H_27_Cl_2_N_2_O_6_Pt^−^ [M-H]^−^ = 715.08783 *m*/*z*, found 715.08929 *m*/*z*, Δ = 2.0 ppm; HESI-HRMS, calcd for C_25_H_29_Cl_2_N_2_O_6_Pt^+^ [M+H]^+^ = 717.10238 *m*/*z*, found 717.10217 *m*/*z*, Δ = −0.3 ppm; ^1^H NMR (DMSO-d_6_) 1.68 (qv, 2H, *J* = 7.3 Hz, CH_2_), 1.97 (qv, 2H, *J* = 7.4 Hz, CH_2_), 2.26 (t, 2H, *J* = 7.4 Hz, CH_2_), 2.27 (t, 2H, *J* = 7.3 Hz, CH_2_), 2.39 (t, 2H, *J* = 7.2 Hz, CH_2_), 6.56 (br. s, 6H, NH_3_), 7.98 (d, 1H, J = 7.9 Hz, HPyr), 8.05 (t, 1H, J = 7.6 Hz, HPyr), 8.11 (d, 1H, J = 9.0 Hz, HPyr), 8.14 (d, 1H, J = 9.0 Hz, HPyr), 8.19–8.23 (m, 2H, HPyr), 8.26 (t, 2H, J = 8.1 Hz, HPyr), 8.46 (d, 1H, J = 9.3 Hz, HPyr), 11.99 (br. s, 1H, COOH); ^195^Pt NMR (DMSO-d_6_) δ: 1226 ppm; IR (ν, cm^−1^): 3560 (br, ν_O-H_), 3245 (s, ν_N-H_), 3132 (s, ν_N-H_), 3035 (s, ν_N-H_), 2940 (m, ν_C-H_), 1706 (s, ν_C=O_), 1647 (vs, ν_C=O_), 1603 (s, ν_C=C_), 1548 (m, ν_C-O_), 1456 (w, ν_C-C_), 1416 (m, ν_C-C_), 1318 m, 1242 m, 1146 (w, δ_C-H_), 840 (vs, γ_C-H_), 668 (m, γ_C-H_).

Complex **4**: (yield, 76 mg; 0.106 mmol; 45.0% relative to Pt). C_24_H_26_Cl_2_N_2_O_7_Pt, MW = 719.076: HESI-HRMS, calcd for C_24_H_25_Cl_2_N_2_O_7_Pt^−^ [M-H]^−^ = 717.06709 *m*/*z*, found 717.06836 *m*/*z*, Δ = 1.8 ppm; HESI-HRMS, calcd for C_24_H_27_Cl_2_N_2_O_7_Pt^+^ [M+H]^+^ = 719.08165 *m*/*z*, found 719.08118 *m*/*z*, Δ = −0.7 ppm; ^1^H NMR (DMSO-*d*_6_) δ: ^1^H NMR (DMSO-d_6_) 1.97 (qv, 2H, *J* = 7.5 Hz, CH_2_), 2.39 (t, 2H, *J* = 7.2 Hz, CH_2_), 4.08 (s, 2H, CH_2_O), 4.10 (s, 2H, CH_2_O), 6.58 (br. s, 6H, NH_3_), 7.98 (d, 1H, J = 7.8 Hz, HPyr), 8.05 (t, 1H, J = 7.6 Hz, HPyr), 8.11 (d, 1H, J = 9.0 Hz, HPyr), 8.14 (d, 1H, J = 9.0 Hz, HPyr), 8.19–8.23 (m, 2H, HPyr), 8.26 (t, 2H, J = 8.0 Hz, HPyr), 8.45 (d, 1H, J = 9.3 Hz, HPyr); ^195^Pt NMR (DMSO-d_6_) δ: 1227 ppm; IR (ν, cm^−1^): 3567 (br, ν_O-H_), 3191 (vs, ν_N-H_), 3093 (s, ν_N-H_), 2972 (m, ν_C-H_), 2926 (m, ν_C-H_), 2876 (m, ν_C-H_), 1729 (s, ν_C=O_) 1653 (vs, ν_C=O_), 1608 (s, ν_C=C_), 1586 (s, ν_C-O_), 1560 (m, ν_C-O_), 1417 (w, ν_C-C_), 1363 m, 1305 s, 1254 s, 1135 (s, δ_C-H_), 846 (s, γ_C-H_).

Complex **5**: (yield, 65 mg; 0.087 mmol; 52% relative to Pt). C_24_H_26_Cl_2_N_2_O_6_PtS, MW = 735.0536: HESI-HRMS, calcd for C_24_H_25_Cl_2_N_2_O_6_PtS^−^ [M-H]^−^ = 733.04425 *m*/*z*, found 733.04608 *m*/*z*, Δ = 2.9 ppm; HESI-HRMS, calcd for C_24_H_27_Cl_2_N_2_O_6_PtS^+^ [M+H]^+^ = 735.05880 *m*/*z*, found 735.05780 *m*/*z*, Δ = −1.4 ppm; ^1^H NMR (DMSO-d_6_) 1.97 (qv, 2H, *J* = 7.6 Hz, CH_2_), 2.39 (t, 2H, *J* = 7.1 Hz, CH_2_), 3.37 (t, 2H, *J* = 7.7 Hz, CH_2_), 3.38 (s, 2H, CH_2_S), 3.40 (s, 2H, CH_2_S), 6.55 (br. s, 6H, NH_3_), 7.99 (d, 1H, J = 7.8 Hz, HPyr), 8.05 (t, 1H, J = 7.6 Hz, HPyr), 8.11 (d, 1H, J = 9.0 Hz, HPyr), 8.14 (d, 1H, J = 9.0 Hz, HPyr), 8.19–8.23 (m, 2H, HPyr), 8.26 (t, 2H, J = 8.0 Hz, HPyr), 8.46 (d, 1H, J = 9.3 Hz, HPyr), 12.5 (very br. s, 1H, COOH); ^195^Pt NMR (DMSO-d_6_) δ: 1228 ppm; IR (ν, cm^−1^): 3500 (br, ν_O-H_), 3197 (vs, ν_N-H_), 3130 (s, ν_N-H_), 3033 (s, ν_N-H_), 2976 (m, ν_C-H_), 1716 (s, ν_C=O_), 1653 (vs, ν_C=O_), 1603 (s, ν_C=C_), 1590 (s, ν_C-O_), 1435 (w, ν_C-C_), 1401 (m, ν_C-C_), 1360 m, 1313 vs, 1213 s, 847 (s, γ_C-H_).

### 2.3. Reduction Experiments

Reductions of complexes **1**–**3** were induced by addition of ascorbic acid (L-AA). The release of the free pyrenebutyric axial ligand was monitored by fluorescence and/or HPLC measurements. The HPLC method was used to monitor the reduction by ascorbic acid (L-AA) of complexes **1**–**3** both in aqueous and non-aqueous solutions for the sake of comparison with the NMR experiments performed in pure DMSO. The more characteristic wavelength at 276 nm was selected for the UV detection of pyrene fragment to avoid interference with the other components in the system. The chromatographic conditions (60:40 ACN: buffer 2.7 (triethylamine-phosphate buffer, TEA-PO_4_)) were optimized so that both the metal complex and the released pyrenebutyric fragment can be detected, with retention times (RT) of 2–5 min and 9 min, respectively. Representative chromatograms are shown in the Supplementary Materials (Figures S26 and S27). The quantitative analysis for the reduction rate of Pt(IV), accompanied by the release of the axial ligands, was performed using a standard solution of the 1-pyrenebutyric acid analyzed before each measurement to avoid fluctuations in the measurements conditions (ambient temperature, etc.). The results are presented as the amount of released 1-pyrenebutyric acid (in %) related to the maximum expected amount in case of total reduction. The monitoring was performed at 0, 3, 6, 12, 24, 54 and 78 h after mixing in a 1:10 ratio of the solutions of the complex and the ascorbic acid, and incubation at 37 °C in the dark. Experimental conditions in aqueous media: 2.9 mg of **2** (0.004 mmol, final concentration 0.412 mM) were dissolved in 0.5 mL DMF. Two mL HEPES buffer (pH = 7), L-AA (7.3 mg, 0.041 mmol) and 7.5 mL ACN were added to a final volume of 10.0 mL. The reaction mixture was sonicated and transferred into two Eppendorf tubes (1.5 mL each) and placed on a hot plate (37 °C). The remaining amount was left in the dark at room temperature. The amount of the released organic ligand was estimated using aliquots of 100 μL of the reaction mixture (after 10-fold dilution with ACN). A control measurement was performed at 170 h after mixing. In the non-aqueous experiments, the following amounts of the complexes were used: 2.2 mg of **1** (0.0036 mmol, final concentration 0.364 mM), 3.2 mg of **2** (0.0045 mmol, final concentration 0.455 mM) and 2.6 mg of **3** (0.0036 mmol, final concentration 0.362 mM) dissolved in 0.5 mL DMF. The 10-fold excess of L-AA was added to the ACN to a final volume of 10.0 mL.

For the fluorescence measurements, stock solutions of compounds **1**–**3** were prepared in DMSO with concentrations of 10^−4^ M. These solutions were used for testing the stability of compounds in the presence of the 10-fold excess of L-AA, dissolved in deionized water. The release of the fluorescent pyrenebutyric ligand was monitored by fluorescence measurements for a period of 3 days.

### 2.4. Cell Lines and Culture Conditions

Human cancer cell lines of leukemic and epithelial origin were used in the present study. The leukemic cell lines BV-173 (BCR–ABL-positive chronic myeloid leukemia) and HL-60 (acute promyelocytic leukemia), as well as the epithelial carcinoma cell lines MDA-MB-231 (triple-negative breast adenocarcinoma), MCF-7 (estrogen-receptor-positive breast adenocarcinoma), and HT-29 (colorectal adenocarcinoma), were cultured under standard conditions. All cell lines were obtained from the German Collection of Microorganisms and Cell Cultures (DSMZ GmbH, Braunschweig, Germany).

Cells were maintained in RPMI-1640 supplemented with 10% fetal bovine serum (FBS), 100 U/mL penicillin, and 100 µg/mL streptomycin. Cultures were incubated at 37 °C in a humidified atmosphere containing 5% CO_2_. Cells were routinely subcultured to maintain logarithmic growth and were used for experiments during the exponential growth phase.

### 2.5. Cytotoxicity Assessment (MTT-Dye Reduction Assay)

The cytotoxic activity of Pt(IV) complexes **2**–**5** and the reference drug cisplatin was evaluated using the MTT colorimetric assay. Cells were seeded in 96-well plates at an appropriate density and allowed to attach overnight under standard culture conditions. Following incubation, cells were treated with the tested compounds over a concentration range of 200.00–3.13 µM, using six serially diluted concentrations. Cells were exposed to the compounds for 72 h. After the incubation period, MTT reagent (3-(4,5-dimethylthiazol-2-yl)-2,5-diphenyltetrazolium bromide) was added to each well and the plates were incubated for an additional 3–4 h to allow formation of formazan crystals by metabolically active cells. The crystals were subsequently dissolved in propanol containing 5% formic acid. Absorbance was measured at 580 nm using a microplate reader (Labexim LMR-1, Allschwil, Switzerland). Cell viability was expressed as a percentage relative to untreated control cells, and IC_50_ values were calculated from dose–response curves obtained from three independent experiments, and using non-linear regression analysis (curve-fit, GraphPad Prizm Software 6.0).

### 2.6. Proteome Profiling of Apoptosis-Related Proteins

To investigate the molecular mechanisms underlying the cytotoxic activity of the Pt(IV) complexes, apoptosis-related protein expression was analyzed using a Proteome Profiler™ Human Apoptosis Array Kit (R&D System Inc., Minneapolis, MN, USA Catalog #ARY009). The most sensitive leukemic model, BV-173, was selected for proteome profiling experiments.

Cells were treated with **3**, **4** or cisplatin at their respective IC_50_ concentrations for 48 h. After treatment, cells were harvested and lysed according to the manufacturer’s protocol. Equal amounts of total protein were incubated with the membrane arrays containing immobilized antibodies against apoptosis-related proteins. Following incubation with detection antibodies and streptavidin-HRP, chemiluminescent signals were developed and recorded using an Azure C600 imaging system (Azure Biosystems C600, Dublin, CA, USA). Protein spots were quantified by densitometric analysis using ImageJ software (version 1.0). Each protein was represented by two duplicate spots, and the mean pixel intensity of the two spots was used for subsequent analysis. Changes in protein expression were evaluated both as absolute signal intensity values and as fold changes relative to untreated control cells.

### 2.7. Acridine Orange/Propidium Iodide (AO/PI) Fluorescence Staining

Morphological changes associated with apoptosis were assessed using acridine orange/propidium iodide (AO/PI) dual fluorescence staining. BV-173 suspension cells were divided into untreated control (Ko) and treatment groups, and incubated with **3**, **4** or cisplatin at their respective IC_50_ concentrations for 48 h. Following treatment, cells were collected, washed with phosphate-buffered saline (PBS), and stained with a mixture of acridine orange and propidium iodide. After staining, cells were washed twice with phosphate-buffered saline (PBS), with centrifugation at 1200 rpm for 5 min between washing steps.

Immediately after resuspension in PBS, cells were mounted on glass slides and examined under a fluorescence microscope. Acridine orange penetrates intact cell membranes and intercalates into DNA, producing green fluorescence in viable cells, whereas propidium iodide enters cells with compromised membrane integrity and stains nucleic acids orange/red, indicating apoptotic or late apoptotic cells. Morphological features such as chromatin condensation, nuclear fragmentation, membrane blebbing, and formation of apoptotic bodies were used to distinguish viable, early apoptotic, and late apoptotic cells.

### 2.8. ICP-MS Analysis of the Platinum Uptake by the Cells and Lipophilicity (logP) Determination

All cell samples were dissolved using 1 mL 67–69% HNO_3_ (Fisher Chemicals, Pittsburgh, PA, USA, Ultra Trace Metal Grade) and left for at least 48 h to achieve complete digestion. Subsequently, the samples were diluted to a final volume of 3 mL and analyzed using ICP-MS. Instrumental measurement was performed for the most abundant natural isotopes—^194^Pt, ^195^Pt and ^196^Pt. Method calibration was performed with standard solutions in the concentration range of 0.5–50 ppb, while ^191^Ir at 20 ppb was used as the internal standard. Calibration curves obtained for the platinum isotopes showed correlation coefficients higher than 0.999 based on linear least squares regression analysis. The detection and quantification limits of the ICP-MS method for platinum analysis were lower than 0.006 ng/mL and 0.018 ng/mL, respectively.

The lipophilicity of the compounds was evaluated using the shake-flask method [36]. Each Pt(IV) complex was dissolved in 0.9% NaCl (*w*/*v*) ultrapure water presaturated with *n*-octanol. The samples were sonicated and filtered to eliminate any insoluble Pt(IV) compounds. Initial platinum levels were quantified by ICP-MS analysis. The aqueous Pt(IV) solutions were then mixed with an equal volume of *n*-octanol previously presaturated with 0.9% NaCl (*w*/*v*). The biphasic mixtures were shaken vigorously for 30 min, followed by centrifugation at 4000 rpm for 30 min to achieve complete separation of the organic and aqueous phases. Platinum concentrations in the aqueous phase were subsequently measured again by ICP-MS. The log*P* values were calculated as the logarithm of the partition ratio of platinum between the organic and aqueous phases.

## 3. Results

### 3.1. Synthesis and Stability of the Complexes

Complexes **2**–**5** were obtained by reacting **1** with 4-fold excess of the corresponding anhydride in DMF, following a published procedure [21]. The disubstituted complexes were readily distinguished from the monosubstituted complex **1** by the absence of the characteristic stretching vibration of the O-H bond at 3481 cm^−1^ in the IR spectrum of **1**. Instead, broad absorption bands are detected at ca. 3500 cm^−1^ and new bands appear at >1705 cm^−1^, which are characteristic for the free carboxylic groups present in the asymmetrically disubstituted complexes **2**–**5** (Figures S1–S5 in the Supplementary Materials). The composition of the complexes was confirmed by HESI-HRMS analyses performed both in positive and negative modes. Molecular ion peaks were confirmed in all cases with high intensity and the detected isotope pattern was in accordance with the theoretically calculated ones (Figures S6–S9 in the Supplementary Materials). The structures of the complexes were further confirmed by NMR spectroscopy (Figures S10–S18 in the Supplementary Materials).

**Figure 2:**
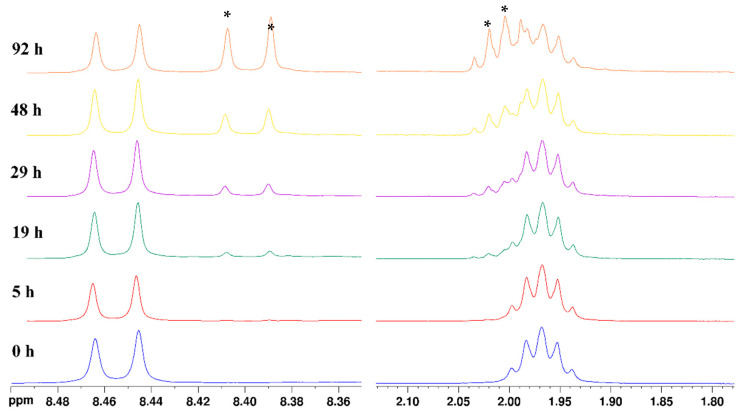
Changes in the ^1^H-NMR spectra upon addition of ascorbic acid to complex **2** (in DMSO-*d*_6_) over 92 h. The appearance of the signals of the free pyrenebutyrate (denoted by asterisks) is shown in two different regions—ca. 8.40 ppm (**left**) and ca. 2.02 ppm (**right**).

**Figure 3:**
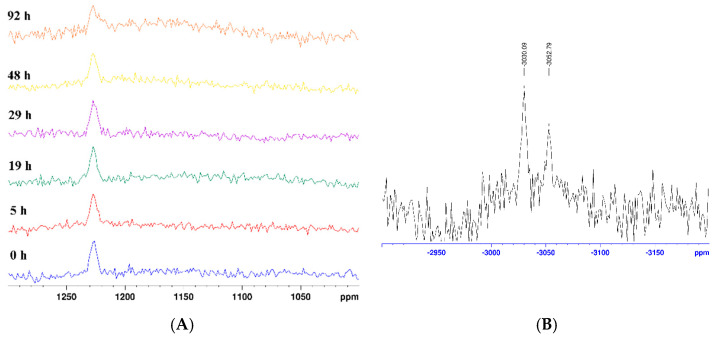
Changes in the ^195^Pt-NMR spectrum of complex **2** after 92 h of adding ascorbic acid (in DMSO-*d*_6_): (**A**) in the 1000 to 1300 ppm region; (**B**) in the −2900 to −3200 ppm region.

The stability of complexes **2**, **3** and **4** was tested over time by NMR spectroscopy. Both ^1^H and ^195^Pt NMR spectra were registered after addition of biological reductants, such as ascorbic acid or glucose (excess of solid powders), to DMSO solutions of complexes **2**, **3** or **4**. The NMR-monitored kinetics of complex **2** revealed that it reacts with ascorbic acid with the reduction of Pt(IV) to Pt(II) and release of the axial ligands. This is evidenced by the changes observed in the ^1^H and ^195^Pt NMR spectra depicted in Figure 2 and Figure 3, respectively. Interestingly, the tested complexes remain intact upon addition of glucose (see Figures S19–S24 in the Supplementary Materials for **3** and **4**).

Over the course of 92 h, complex **2** partially releases the pyrenebutyrate axial ligand upon reaction with ascorbic acid. This was evidenced by the changes in the aromatic region of the ^1^H NMR spectrum, presented in Figure 2. The signals for the liberated pyrenebutyrate appear at ca. 8.40 ppm after 19 h. Similar changes are observed in the aliphatic region of the spectrum with the appearance of new signals at 2.02 ppm. The reduction of complex **2** is confirmed by the loss of the characteristic NMR signal for Pt(IV) nuclei at 1227 ppm (Figure 3). After a scrutinized search in broad ^195^Pt NMR spectral ranges (from 3000 to −4000 ppm), the signals of the formed Pt(II) species of the reduced complex **2** were detected at −3030 and −3052 ppm. The signal for the Pt(IV) species, however, does not disappear after 92 h, which confirms again the partial reduction of complex **2** to its Pt(II) analogue. The two signals in the ^195^Pt NMR of the formed Pt(II) species most probably correspond to different coordination modes of the DMSO molecules in the formed complexes (either with the S- or O-atom). More details on the NMR shielding of the Pt nuclei in Pt(II) complexes with DMSO have been discussed earlier by other authors [37].

**Figure 4:**
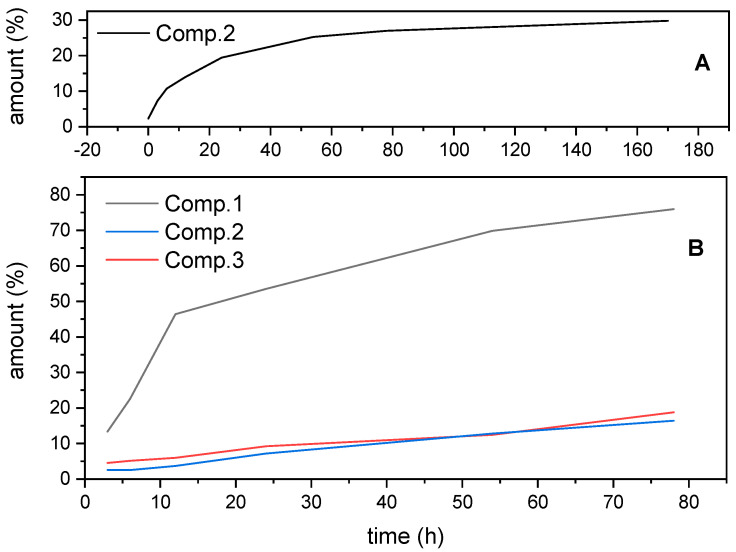
Reduction of complexes **1**–**3** by ascorbic acid upon incubation in 10-fold excess for 3 days: (**A**) in HEPES buffer; (**B**) in non-aqueous media.

Due to the limited sensitivity of the NMR method, which usually requires a higher amount of the studied compounds, further studies on the reduction profiles of the complexes by 10-fold excess of ascorbic acid was performed using HPLC and fluorescence methods. For the sake of comparison with the NMR kinetics available for compounds **1** [14] and **2**, measurements were performed in non-aqueous media over the course of 3 days (76 h more precisely). The amounts of the released-by-reduction pyrenebutyric fragment estimated from the HPLC experiments are 75.9% for compound **1**, but only 16.4 and 18.8% for compounds **2** and **3**, respectively (Figure 4B). Notably, the experiment in HEPES buffer (and pH 7) increases the reduction rate of compound **2** almost twice (to 27%, Figure 4A). A similar trend is observed from the fluorescence measurements of the liberated pyrene fragment upon reduction by L-AA in DMSO–water medium (Figure S25 in the Supplementary Materials).

### 3.2. Cytotoxicity Screening Results

The antiproliferative activity of the compounds in the studied series was evaluated against a panel of human malignant cell lines of both leukemic (BV-173, HL-60) and epithelial origin (MDA-MB-231, MCF-7, HT-29), and compared with the parent Pt(II) complex, cisplatin. The cytotoxicity results presented in Table 1 reveal clear trends in potency across compounds and cell models, providing insight into the influence of the axial ligand structure on the biological activity of the Pt(IV) cisplatin analogues.

The estimated cytotoxicity of the studied Pt(IV) complexes reveals clearly superior activity compared to cisplatin. Among all tested compounds, **3** emerges as the most potent analogue across the entire spectrum of cell lines, displaying micromolar to submicromolar IC_50_ values, far exceeding the potency of the reference drug in every tested model, often by nearly an order of magnitude. Its activity is particularly pronounced in the leukemic BV-173 model, where an IC_50_ of 0.19 µM is observed, representing a more-than-25-fold increase in potency compared with cisplatin (5.2 µM). Similarly strong effects were observed against HL-60 leukemic cells (3.9 µM vs. 8.4 µM for cisplatin) and all epithelial cancer models, i.e., MDA-MB-231 (2.1 µM vs. 55.7 µM for cisplatin) and MCF-7 (10.0 µM vs. 50.5 µM). Notably, complex **3** was the only analogue that achieved substantial cytostatic activity in the cisplatin-resistant colorectal carcinoma cell line HT-29, exhibiting an IC_50_ value of 14.4 µM compared with 117.8 µM for the reference drug. In accordance with the reduction kinetics data presented in Figure 4B, complex **1** exhibits the highest cytotoxicity against all tested cancer cells, which clearly correlates with its fastest reduction by ascorbic acid.

Complex **4** also exerted broad spectrum antitumor activity, with a potency slightly inferior to that of **3** in most models, but nevertheless markedly greater than that of cisplatin. In BV-173 cells, **4** exhibited an IC_50_ of 0.3 µM, affirming the exceptional sensitivity of this leukemic model. Potent growth inhibition was also observed in HL-60 (3.5 µM), MDA-MB-231 (9.6 µM) and MCF-7 (12.4 µM) cells, while HT-29 colorectal carcinoma cells displayed somewhat reduced susceptibility (38.3 µM).

Complex **5** demonstrated intermediate activity across most models, with IC_50_ values ranging between 4 and 13 µM in BV-173, HL-60, and MDA-MB-231 cells, and moderately weaker activity in the cisplatin-resistant HT-29 cell line (35.7 µM). Nevertheless, its potency remains consistently superior to that of cisplatin in all models. Notably, all Pt(IV)-pyrenebutyrate analogues in the series outperformed the reference Pt(II) drug in the screening experiments with HT 29 cells, achieving approximately 3- to 4-fold-lower IC_50_ values.

Complex **2** and the free ligand (1-pyrenebutyric acid) were tested against MCF-7 and HT-29 cells. This Pt(IV) complex exhibited relatively strong cytostatic activity in MCF-7 and HT-29 cells, (IC_50_ values of 12.3 and 29.7 µM, respectively). It is somewhat less active against the HL-60 cells as compared to the other three complexes (IC_50_ value of 12.1 µM vs. 8.4 µM for cisplatin). Notably, the free ligand **L** showed limited activity in HT-29 (>200 µM) and MCF-7 cells (112 µM), indicating that it is not toxic to these particular cell lines. Slightly higher cytotoxicity was found, however, against HL-60 cells, which is in accordance with the known higher susceptibility of this cell line.

### 3.3. Platinum Uptake and Lipophilicity

**Figure 5:**
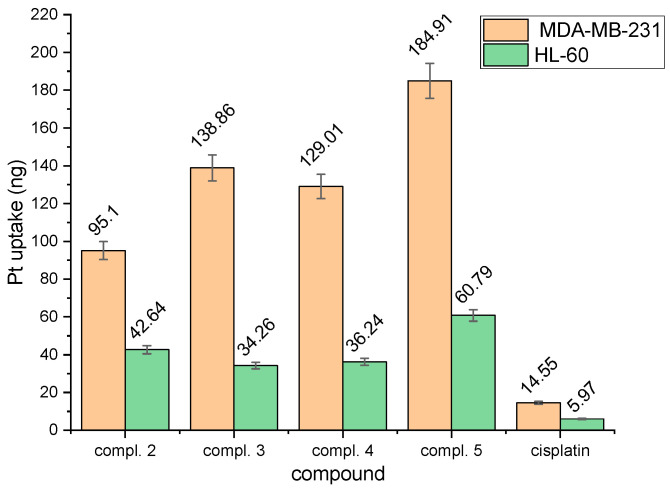
Pt uptake (in ng Pt/10^6^ cells) by the HL-60 and MDA-MB-231 cells after 4 h of incubation with 10 μM solutions of complexes **2**–**5** and cisplatin.

In search of explanations for the observed trends in the estimated cytotoxicity of the studied complexes, we measured the platinum uptake by two types of cancer cells HL-60 and MDA-MB-231, which are of suspension and adherent types, respectively. The cells were incubated for 4 h with 10 μM solutions of all Pt(IV) complexes and cisplatin The data were obtained from ICP-MS measurements and are presented in Figure 5 as Pt content (in ng) per 1 million cells.

Among all tested compounds, complex **5** shows the highest platinum uptake by MDA-MB-231 cells, which prevails more than 10 times the uptake from cisplatin treatment and almost twice that of complex **2**. In the case of HL-60 cells, the Pt uptake after treatment with the Pt(IV) complexes follows the same trend and prevails almost 10 times that of cisplatin treatment. Generally, these data correlate very well with the cytotoxicity results on these cell lines with only few exceptions regarding the lower IC_50_ value of complex **2** in HL-60 cells, despite its high uptake, and the lower IC_50_ value of complex **5** in MDA-MB-231 cells despite the highest uptake it shows compared to all other complexes. Such a trend, as seen in our previously published uptake data on monosubstituted and symmetrically disubstituted complexes [14], was explained with the reduction kinetics of both types of complexes showing the crucial effect of the structural modifications.

Clearly, further factors related to the fate of the complexes after internalization are operative for their physiological activity, which may comprise their chemical stability, physicochemical characteristics and the complicated interaction with an array of proteins interfering with their expression [38]. As a measure of the lipophilicity of the complexes, we estimated the *n*-octanol/water partition coefficient log*P*, and the data are summarized in Table 2. All complexes show moderate lipophilicity, which is less pronounced for complexes **3** and **4**. The latter are the complexes with the highest cytotoxicity in the studied series of complexes, and their mechanism of action was further elaborated with proteome analysis.

### 3.4. Proteome Profiling Results

**Figure 6:**
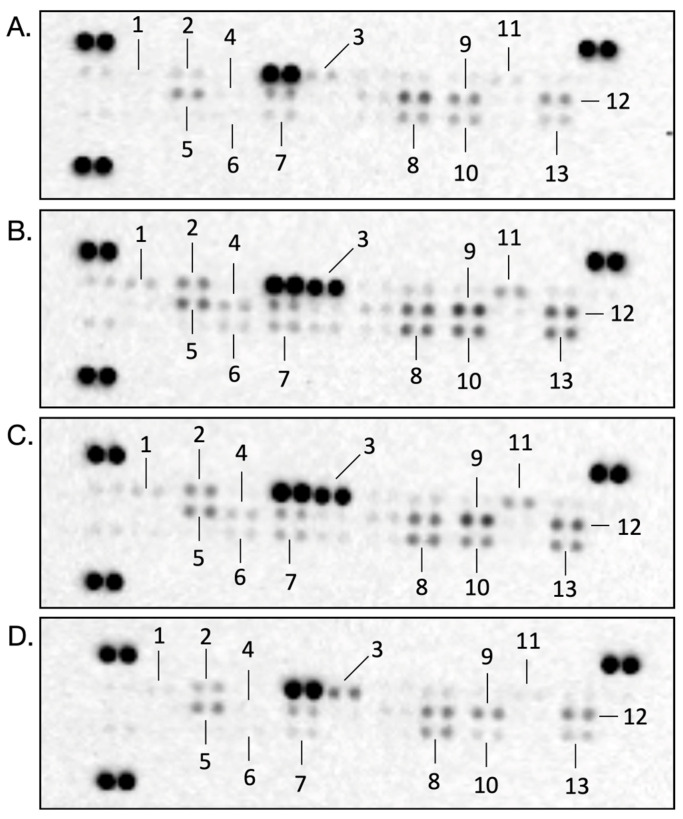
Representative membrane arrays, showing differential expression of apoptosis-related proteins in BV-173 cells (Proteome Profiler™ Human Apoptosis Array Kit, R&D Systems, Catalog #ARY009). Panel (**A**) indicates the untreated control sample (Ko), while panels (**B**–**D**) correspond to cells treated with equi-effective concentrations (IC_50_) of **4** (**B**), **3** (**C**), and cisplatin (**D**) for 48 h. Spot intensity reflects the relative abundance of the detected proteins, which were subjected to a densitometric analysis and quantification using ImageJ software (version 1.0). Protein positions are indicated as follows: 1—Bax; 2—Bcl-2; 3—Cleaved Caspase-3; 4—Fas/TNFRSF6/CD95; 5—FADD; 6—Phospho-p53 (S15); 7—Phospho-p53 (S46); 8—SMAC/Diablo; 9—HSP70; 10—Survivin; 11—Claspin; 12—HTRA2/Omi; 13—XIAP.

**Figure 7:**
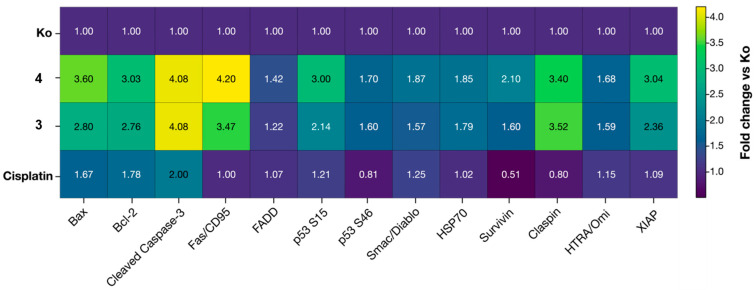
Heatmap representations of **3**-, **4**- and cisplatin-induced changes in protein expression, normalized to untreated control cells (Ko) and based on the mean pixel density values of the duplicate spots corresponding to each protein on the membrane array, determined by ImageJ densitometric analysis after 48 h exposure at IC_50_ concentrations.

Proteome profiling of BV-173 cells treated with the two most potent Pt(IV) derivatives in the series, **3** and **4**, provides important mechanistic insights into their antileukemic activity (Figure 6 and Figure 7). BV-173 was selected for this analysis as it was the most sensitive leukemic model in the biological screening, enabling evaluation of apoptotic signaling events associated with compound-induced cell death. The differential expression of apoptosis-related proteins across samples (Figure 6) was analyzed both in terms of absolute pixel density values derived from densitometric quantification (Figure S26) and as fold changes relative to the untreated control (Figure 7). Overall, comparison of expression profiles across treatment groups revealed a coordinated activation of both intrinsic and extrinsic apoptotic signaling pathways, accompanied by compensatory survival mechanisms triggered in response to DNA damage and cellular stress. When compared with the reference drug cisplatin, both **3** and **4** induced stronger and more coherent proapoptotic responses, suggesting that these Pt(IV) derivatives trigger apoptosis through a broader and more efficient network of signaling events.

### 3.5. Results from the AO/PI Staining Assay

Fluorescence microscopy analysis using acridine orange/propidium iodide (AO/PI) staining was performed to visualize morphological changes associated with apoptosis in BV-173 leukemic cells following treatment with the most potent Pt(IV) derivatives, **3** and **4**, in comparison with the reference drug, cisplatin. This dual staining method allows discrimination between viable and apoptotic cells based on membrane integrity and nuclear morphology. Acridine orange penetrates intact cell membranes and intercalates into DNA, producing green fluorescence in viable cells, whereas propidium iodide enters cells with compromised membranes and binds nucleic acids, resulting in the orange-to-red fluorescence characteristics of apoptotic or late apoptotic cells.

**Figure 8:**
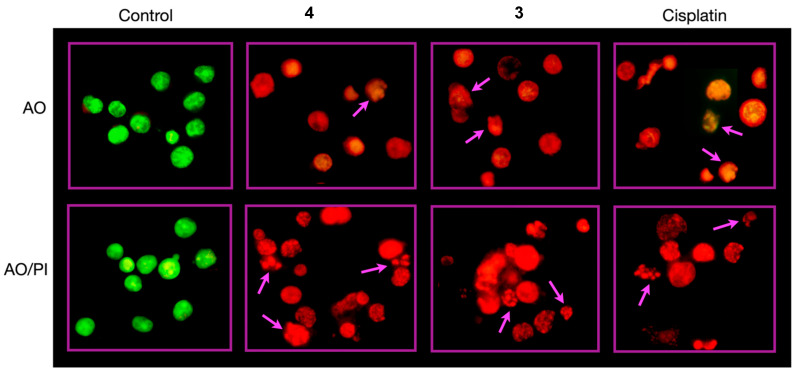
Fluorescence microscopy detection of apoptosis in BV-173 cells following treatment with Pt(IV) complexes **3** and **4** compared with cisplatin. Samples were stained with AO/PI following 48 h exposure to the tested compounds at IC_50_ concentrations. Arrows indicate apoptotic cells, consistent with the activation of apoptotic pathways detected in the proteome profiling experiments.

In the untreated control group, BV-173 cells displayed a typical viable phenotype characterized by round nuclei with uniform green fluorescence, indicating intact membranes and normal chromatin organization (Figure 8). No significant morphological abnormalities were observed, and the cells maintained a homogeneous appearance consistent with healthy proliferating leukemic cells.

In contrast, all treatment groups (**3**, **4** and cisplatin) produced pronounced morphological changes consistent with apoptosis (Figure 8). A substantial proportion of cells exhibited intense orange-to-red fluorescence, reflecting loss of membrane integrity and advanced stages of programmed cell death. In addition to the color shift in fluorescence, clear structural alterations were evident, including membrane blebbing, chromatin condensation, nuclear fragmentation, and the formation of apoptotic bodies. The presence of these hallmark apoptotic structures indicates active execution of the apoptotic program and complements the molecular findings obtained from the apoptosis proteome profiling experiments.

## 4. Discussion

In this study, we expand our earlier results on mono- and disubstituted pyrenebutyrate Pt(IV) complexes, which showed rather distinct reactivity and anticancer activity [14]. To finetune these characteristics, we varied the type of the axial ligands and monitored their effect. The new series of complexes (**2**–**5**) was obtained following earlier reported procedures for synthesis of asymmetric Pt(IV) complexes derived from cisplatin [21,39]. The pyrene fragment was chosen as a fluorescent tag that is also able to intercalate to DNA molecules [40]. Notably, pyrene is a potent guest molecule for probing host–guest interactions with other aromatic systems [32,33,34] that we tested for their anticancer activity, which showed that pyrene [32] and the pyrenebutyrate derivatives [33,34] do not exhibit intrinsic cytotoxicity. Herein, except for the intercalating pyrenebutyrate, we attached different carboxylates to monitor the lipophilicity and the reduction kinetics of the formed Pt(IV) complexes (**2**–**5**), as well as their anticancer potency. All complexes were isolated in good yields and purity, and their structures were confirmed by HRESI-MS, multinuclear NMR spectra and IR spectroscopy (all shown in the Supplementary Materials). Similarly to compound **1** [14], the initial reduction experiments followed by ^1^H and ^195^Pt NMR indicated that only the ascorbic acid, not the glucose, can reduce complexes **2** and **3**. The NMR data (Figure 2 and Figure 3) were complemented by the more sensitive HPLC and fluorescence measurements that were performed for complexes **1**–**3** upon addition of the excess of ascorbic acid (Figure 4 and Figure S25, respectively). The results show that unlike the monosubstituted complex **1**, the asymmetrically disubstituted complexes **2** and **3** liberate the pyrenebutyric fragment upon reduction of the platinum center to Pt(II) only partially over the course of 3 to 5 days. The HPLC data showed that the reduction of complexes **2** and **3** is 4.75 and 4 times less than that of complex **1** under the same experimental conditions (non-aqueous medium). In HEPES buffer, the reduction rate increases twice but remains lower than 1/3 of the expected for total reduction (Figure 4A). The more pronounced reactivity of the monosubstituted Pt(IV) complex **1** agrees with other observations [21,41]; however, the role of the equatorial ligands may also be operative [22,23]. The reduction rates of complexes **1**–**3** was conveniently monitored by fluorescence measurements due to the intrinsic characteristic emission of the liberated pyrene fragment, which is efficiently quenched when coordinated to the Pt(IV) centers due to the heavy element effect. The fluorescence data confirmed the HPLC and NMR observations of only partial reductions of the asymmetrically substituted complexes **2** and **3**, and being as much as twice as lower than that of complex **1** (Figure S25). In this way, by using three independent methods, we could monitor the efficiency of the ascorbic acid to reduce the studied Pt(IV) complexes in different media and as a prerequisite to the activation-by-reduction mechanism of the cytotoxic action of the Pt(IV) prodrugs.

We compared the lipophilicity of all five complexes as estimated from the *n*-octanol–water partition by shake-flask method and ICP-MS measurements. All five complexes are lipophilic, which is more pronounced for complexes **1**, **2** and **5**. Slightly lower is the lipophilicity of the complexes bearing the glutaric and diglycolic fragments, complexes **3** and **4**, respectively. Interestingly, these two complexes also show slightly lower internalization in the suspension-growing HL-60 cells, as obtained from the ICP-MS measurements of the platinum uptake. Their internalization in the adherent cell line, however, exceeds by almost 50% the uptake of complex **2** (Figure 4). Comparison with the uptake data of complex **1**, which is reported to be 5-fold higher than cisplatin in HL-60 cells and 15-fold higher in the HT-29 cells (Table S1 from [14]), demonstrates the distinct uptake mechanisms for cellular internalization in the adherent and suspension-growing cell lines going beyond simple lipophilicity-driven passive transport. The most lipophilic complex **5** shows the highest platinum uptake in both types of tested cells, HL-60 and MDA-MB-231. The potency of cell penetration of specifically pyrenebutyrate modified peptides has already been demonstrated [42], and could also be the reason for the observed very high penetration rate of complexes **1**–**5** as compared to cisplatin—mostly an order-of-magnitude-higher uptake of the studied Pt(IV) complexes.

Collectively, the observed trends in the reactivity, lipophilicity, and platinum uptake by the cancer cells of all studied compounds is expected to give an impact of their cytotoxicity profiles. The cytotoxicity screening reveals that all tested Pt(IV) complexes (particularly **3**, **4**, and **5**) demonstrate markedly superior antitumor activity compared to cisplatin across a range of cancer cell lines, including models with intrinsic resistance. When compared to the monosubstituted complex **1** reported earlier [14], however, they exhibit lower toxicity, which is consistent with the lower rate of reduction of the asymmetrically disubstituted complexes by the biological reductant, ascorbic acid.

With submicromolar IC_50_ values in BV-173 (0.19 µM) and low micromolar activity in HL-60 (3.9 µM), MDA-MB-231 (2.1 µM), and MCF-7 (10.0 µM), complex **3** outperforms cisplatin by nearly one order of magnitude or more. Most striking is its effect in the cisplatin-resistant HT-29 colorectal line (14.4 µM vs. 117.8 µM for cisplatin), indicating that **3** can overcome cross-resistance mechanisms that severely limit the reference drug. This suggests a distinct cytotoxic mechanism or improved cellular accumulation, which was proved by our experiments. Complex **4** also exhibits strong growth inhibition, particularly in BV-173 (0.3 µM) and HL-60 (3.5 µM), and remains far more effective than cisplatin in epithelial models (MDA-MB-231: 9.6 µM vs. 55.7 µM; MCF-7: 12.4 µM vs. 50.5 µM). Its reduced efficacy in HT-29 (38.3 µM) compared to complex **3** suggests that structural modifications between **3** and **4** critically influence activity in resistant settings. These two complexes are the most cytotoxic ones in the studied series of asymmetrically disubstituted Pt(IV); therefore, they were further subject to proteome analysis. Their distinct lower lipophilicity (Table 2) may also play a role in their accumulation in different cellular milieu, thereby facilitating the interaction with the bioavailable reductants. A common structural feature of these two complexes is that they possess a carboxylate fragment of the same length as the second axial ligand. Seemingly, the replacement of an CH_2_ group (in complex **3**) with an ether O-atom (in complex **4**) does not strongly affect their cytotoxicity and cellular internalization. On the contrary, replacement of the same CH_2_ group with a thioether S-atom in complex **5** results in appreciably higher lipophilicity and the highest-measured platinum uptake by both suspension-growing and adherent cells, as compared with all other tested complexes. Complex **5** achieves IC_50_ values of 4–13 µM in most sensitive lines and remains 3- to 4-fold more potent than cisplatin in HT-29 (35.7 µM vs. 117.8 µM). Complex **2**, tested only in MCF-7 and HT-29, shows notable activity (12.3 µM and 29.7 µM, respectively) but is less effective in HL-60 (12.1 µM vs. 8.4 µM for cisplatin). Importantly, the free ligand (1-pyrenebutyric acid) is essentially non-toxic in HT-29 (>200 µM) and MCF-7 (112 µM), confirming that the Pt(IV) core is essential for the observed cytotoxicity.

The data from the proteome analysis on complexes **3** and **4** highlight several pro-apoptotic mechanisms that can be outlined as follows:

Intrinsic (Mitochondrial) Apoptotic Signaling

A central determinant of mitochondrial apoptosis is the balance between pro- and anti-apoptotic members of the Bcl-2 protein family, which regulate mitochondrial outer membrane permeabilization. In this context, treatment of BV-173 cells with both Pt(IV) complexes **3** and **4** resulted in a pronounced increase in Bax expression (3.6- and 2.8-fold, respectively), indicating a clear shift toward pro-apoptotic signaling. Bax is a key mitochondrial effector that promotes membrane permeabilization and the release of apoptogenic factors such as cytochrome c, thereby initiating downstream caspase activation. In comparison, cisplatin induced only a moderate increase in Bax (~1.7-fold), suggesting a less efficient activation of mitochondrial apoptosis under the same conditions.

Interestingly, expression of the anti-apoptotic protein Bcl-2 was also elevated following treatment with both Pt(IV) derivatives (approximately 3-fold). As Bcl-2 antagonizes Bax-mediated mitochondrial permeabilization, its induction likely reflects a compensatory cellular response attempting to counteract excessive mitochondrial damage. Such adaptive upregulation of anti-apoptotic proteins is commonly observed in cells exposed to strong cytotoxic stress [43]. Nevertheless, the substantially greater increase in Bax suggests that the overall Bax/Bcl-2 balance remains shifted toward apoptosis in all treated groups.

Further evidence for activation of mitochondrial apoptotic signaling is provided by the increased expression of SMAC/DIABLO and HTRA2/Omi, two mitochondrial proteins released upon mitochondrial membrane permeabilization. SMAC/DIABLO expression increased approximately 1.9-fold following complex **4** treatment and slightly less after **3** exposure, whereas cisplatin produced a more modest induction (1.25-fold). HTRA2/Omi also showed preferential upregulation (~1.6–1.7-fold) in the Pt(IV)-treated samples compared to the referent complex, cisplatin (1.15-fold). Both proteins promote apoptosis by antagonizing members of the IAP family (inhibitor of apoptosis proteins) and facilitating caspase activation. The coordinated increase in Bax, SMAC/DIABLO, and HTRA2/Omi therefore strongly indicates a more robust activation of the intrinsic mitochondrial apoptotic pathway by the Pt(IV) derivatives.

Extrinsic Apoptotic Signaling

In addition to mitochondrial apoptosis, the proteomic data also indicate enhanced activation of the extrinsic, death-receptor-mediated pathway. Fas/CD95, a cell surface death receptor that initiates apoptotic signaling upon ligand binding, was strongly induced following treatment with complexes **3** (~3.5-fold) and **4** (~4.2-fold), whereas cisplatin exposure did not produce statistically significant changes in its levels.

Notably, the expression profile of the FADD adaptor protein engaged in the extrinsic apoptotic pathway also showed a modest induction following treatment with complexes **3** and **4** (1.42- and 1.22-fold, respectively). In contrast, FADD levels in the reference cisplatin group showed only a marginal increase of 7%. In concert with the observed upstream activation of Fas/CD95, the recruitment of FADD facilitates activation of initiator caspase-8 and subsequent propagation of the apoptotic cascade, supporting the involvement of death receptor signaling in the mechanistic behavior of the Pt(IV) species. In addition, the pronounced upregulation of Bax observed in Pt(IV)-complex-treated cells may further amplify this signaling, given its role as a molecular link between the extrinsic and intrinsic apoptotic pathways.

Caspase Activation and Execution of Apoptosis

The most striking change in the proteomic profile across samples was the strong induction of cleaved caspase-3, which increased approximately 4-fold following treatment with both complexes **3** and **4**. The strong simultaneous activation of both intrinsic and extrinsic apoptotic pathways likely contributes to the pronounced activation of the downstream executioner caspase, preferentially observed in the Pt(IV)-exposed BV-173 cells. Cleaved caspase-3 represents the active form of the enzyme and serves as the central executioner of apoptosis, responsible for the proteolytic cleavage of numerous cellular substrates leading to the characteristic morphological and biochemical features of programmed cell death. Notably, treatment with both Pt(IV) derivatives resulted in maximal activation of caspase-3 (as evidenced by the maximal signal intensity detected in the corresponding array spots), clearly exceeding the magnitude of response observed with cisplatin (a ca. 2-fold increase in the protein levels). These findings are consistent with the elevated expression and mitochondrial release of SMAC/DIABLO, which further promotes caspase activation in the **3**- and **4**-treated groups.

p53 Signaling and DNA Damage Response

Further evidence for apoptosis induction by the Pt(IV) analogues is provided by the functional status of the p53 signaling pathway. Phosphorylation of p53 at Ser15 is substantially higher with complexes **3** (~2.1 fold) and **4** (~3 fold) than with cisplatin (~1.2 fold), indicating more effective stabilization and activation of p53 in response to Pt(IV)-induced genotoxic stress. Additional phosphorylation at Ser46, a modification associated with transcriptional activation of pro-apoptotic p53 target genes, was also moderately increased following treatment with complexes **3** (1.6-fold) and **4** (1.7-fold), further supporting a shift toward irreversible cell death signaling. Notably, cisplatin did not promote activation of p53 at this regulatory site and instead resulted in an approximately 20% reduction in phosphorylation at Ser46 of the tumor suppressor protein.

Cell Cycle Regulation and Stress Response Pathways

Proteins involved in stress responses and cell cycle regulation were also modulated. HSP70 expression increased moderately following treatment with complexes **3** and **4** (~1.8-fold). HSP70 is a stress-inducible molecular chaperone that maintains protein homeostasis under conditions of cellular stress, including oxidative or proteotoxic damage. Its induction likely reflects activation of a protective stress response triggered by the Pt(IV) derivatives. In contrast, cisplatin did not induce significant changes in HSP70 expression levels compared to the control group.

In parallel, claspin, a key mediator of ATR-Chk1 signaling, is strongly induced following treatment with both Pt(IV) compounds (3.4–3.5-fold). As an adaptor protein in the DNA damage response, claspin participates in checkpoint signaling and functions as an anti-apoptotic ‘brake’, promoting cell cycle arrest and delaying apoptotic progression under conditions of genotoxic stress. Its pronounced upregulation therefore likely represents a compensatory protective response aimed at counteracting the strong pro-apoptotic signaling triggered by the Pt(IV) derivatives. The marked induction of claspin is therefore consistent with substantial replication stress and supports the notion that these Pt(IV) complexes may generate more persistent or structurally complex DNA lesions than cisplatin.

Compensatory Anti-Apoptotic Responses

Despite the strong pro-apoptotic signaling pattern, several anti-apoptotic proteins were also upregulated. Survivin and XIAP, two prominent members of the inhibitor-of-apoptosis-protein family, showed increased expression following treatment with the Pt(IV) derivatives. Survivin levels increased approximately 2.1-fold after treatment with complex **4** and 1.6-fold following exposure to complex **3**, whereas cisplatin caused a reduction in survivin expression (~0.5-fold). Similarly, XIAP expression increased approximately 2.4-fold with complex **3** and 3-fold with complex **4**. Both proteins act as key regulators of cell survival by inhibiting caspase activity and supporting mitotic progression. Their upregulation likely reflects a compensatory response to strong apoptotic signaling. However, the concurrent increase in SMAC/DIABLO may counteract XIAP-mediated caspase inhibition, as SMAC binds and neutralizes XIAP, thereby restoring caspase activity. Consequently, although survivin and XIAP induction indicates activation of survival pathways, these responses appear insufficient to overcome the sustained pro-apoptotic signaling triggered by complexes **3** and **4**.

Collectively, the proteomic data indicate that complexes **3** and **4** trigger a multifaceted apoptotic program in the leukemic BV-173 model that is both stronger and more integrated than that elicited by cisplatin. Both Pt(IV) analogues concurrently engage intrinsic and extrinsic pathways, amplify mitochondrial pro-apoptotic signaling and more effectively activate p53-dependent DNA damage responses, culminating in maximal caspase-3 activation. Although compensatory stress and survival mechanisms (Bcl-2, survivin, XIAP, HSP70, and claspin) were more strongly induced following Pt(IV) exposure, their protective effects appear to be functionally outweighed by Bax/SMAC/HTRA2-mediated mitochondrial permeabilization and pronounced activation of executioner caspases, which was less evident in the cisplatin-treated group. These findings are in line with the superior cytotoxic potency of complexes **3** and **4** in BV 173 cells and support their further exploration as Pt(IV)-based anticancer candidates.

The microscopic observation of apoptotic morphology in all treated groups (Figure 8) confirms that both Pt(IV) complexes and cisplatin successfully execute the cell death program. However, when connected to the molecular data, it becomes clear that complexes **3** and **4** achieve this endpoint through a more aggressive and coordinated mechanism: they simultaneously attack via mitochondrial, death receptor, and p53 pathways, leading to maximal caspase-3 activation. The fact that the morphological changes are pronounced in their presence indicates that the pro-apoptotic signal driven by complexes **3** and **4** is sufficiently powerful to overwhelm cellular defenses and drive the cell to the characteristic, irreversible morphological features of apoptosis.

## 5. Conclusions

This study expands on previous work with mono- and disubstituted pyrenebutyrate Pt(IV) complexes with a series of four new asymmetrically disubstituted complexes (**2**–**5**). The pyrene fragment serves as a fluorescent DNA intercalator and a probe for host–guest interactions, while the pyrenebutyrate itself lack intrinsic cytotoxicity. Different carboxylate axial ligands were introduced to tune lipophilicity, reduction kinetics, and anticancer properties. Reduction experiments using ascorbic acid (but not glucose) showed that the asymmetrically disubstituted complexes **2** and **3** reduce to a lower extent than the monosubstituted complex **1**. NMR, HPLC, and fluorescence measurements consistently indicated that complexes **2** and **3** undergo only partial reduction (over 3–5 days), with reduction rates 4–5 times lower than that of complex **1** in non-aqueous medium, and still below one-third of total reduction in HEPES buffer.

Lipophilicity (measured by octanol–water partition) was high for all complexes, especially for **1**, **2**, and **5**, with complexes **3** and **4** being slightly less lipophilic. Cellular internalization in HL-60 and MDA-MB-231 cells, measured by platinum uptake, was highest for the most lipophilic complex **5**. Notably, all Pt(IV) complexes exhibited approximately an order-of-magnitude-higher cellular uptake than cisplatin. In adherent cells, complexes **3** and **4** showed nearly 50%-higher uptake than complex **2**. The high penetration is attributed to the pyrenebutyrate moiety, known to enhance cell entry.

The cytotoxicity data clearly show that all tested Pt(IV) complexes, especially **3** and **4**, are markedly more potent than cisplatin across multiple cancer models. The observed superior potency in the BV-173 cells of complexes **3** and **4**, which are >25-fold and >17-fold more potent than cisplatin, correlates with robust multi-pathway apoptosis. Activity in cisplatin-resistant HT-29 cells reflect alternative death signaling, which was evidenced for complex **3**, while complexes **4** and **5** were less effective but still superior to cisplatin. Limited cytotoxicity of the ligand alone (IC_50_ >200 µM in HT-29, 112 µM in MCF-7) proves that the observed cell death is not due to the organic moiety per se, but rather requires the intact Pt(IV) complex to deliver pro-apoptotic signaling. While still more potent than cisplatin, complexes **5** (IC_50_ range 4–13 µM) and **2** (e.g., 12.3 µM in MCF-7) did not reach the submicromolar potency of **3** and **4**.

The proteome analysis on the most cytotoxic Pt(IV) complexes (**3** and **4**) indicated their potency to induce apoptosis in BV-173 cells through a stronger and more integrated mechanism than cisplatin. They robustly activate the intrinsic (mitochondrial) pathway by increasing the pro-apoptotic protein Bax (3.6- and 2.8-fold) and releasing SMAC/DIABLO and HTRA2/Omi, shifting the Bax/Bcl-2 balance toward cell death. Simultaneously, they engage the extrinsic death receptor pathway by upregulating Fas/CD95 and FADD. This dual activation leads to a marked 4-fold increase in cleaved caspase-3, the central executioner of apoptosis, far exceeding the effect of cisplatin. The Pt(IV) complexes also enhance pro-apoptotic p53 signaling (Ser15 and Ser46 phosphorylation). Although compensatory anti-apoptotic responses (Bcl-2, survivin, XIAP) are observed, they are functionally outweighed by the pronounced pro-apoptotic signaling, resulting in superior apoptotic potency compared to cisplatin.

In conclusion, the cytotoxicity rankings (**3** > **4** > **5** ≈ **2** >> cisplatin) directly mirror the molecular hierarchy of apoptosis induction. The exceptional potency of complex **3**, especially in resistant models, is best explained by its ability to activate simultaneously and robustly intrinsic, extrinsic, and p53-dependent apoptotic pathways, overwhelming compensatory survival signals. By systematically varying the axial ligands of the Pt(IV) octahedral center, we demonstrate a synthetic approach to finetune key physicochemical and pharmacological parameters, providing a roadmap for designing next-generation anticancer agents with superior potency and pharmacological profiles.

## Figures and Tables

**Table 1:** In vitro cytotoxicity [IC_50_ µM ± SD] of Pt(IV) complexes **1**–**5**, compared with the 1-pyrenebutyric acid **L** and cisplatin against a panel of human malignant cell lines of different origin.

Cell Line/ Compound	HL-60 ^a^	BV-173 ^b^	MDA-MB-231 ^c^	MCF-7 ^d^	HT-29 ^e^
**1** *	0.07 ± 0.01	n.d.	0.7 ± 0.2	0.3 ± 0.15	3.4 ± 0.7
**2**	12.1 ± 0.9	n.d.	n.d.	12.3 ± 2.0	29.7 ± 3.3
**3**	3.9 ± 0.3	0.19 ± 0.01	2.1 ± 0.5	10.0 ± 1.3	14.4 ± 2.7
**4**	3.5 ± 0.2	0.3 ± 0.04	9.6 ± 1.2	12.4 ± 1.5	38.3 ± 4.8
**5**	5.9 ± 1.3	4.1 ± 0.9	13.0 ± 1.5	8.9 ± 1.9	35.7 ± 5.6
**L**	80.1 ± 7.8	n.d.	n.d.	112.1 ± 12.2	>200
cisplatin	8.4 ± 1.6	5.2 ± 0.6	55.7 ± 5.7	50.5 ± 5.3	117.8 ± 7.1

^a^ human acute promyelocytic leukemia; ^b^ human chronic myeloid leukemia, BCR-ABL positive; ^c^ human breast adenocarcinoma, triple-negative; ^d^ human breast adenocarcinoma, estrogen-receptor-positive; ^e^ human colorectal adenocarcinoma. n.d.—not determined. * data from [14].

**Table 2:** Lipophilicity of Pt(IV) complexes **1**–**5** as obtained from the ICP-MS measurements of octanol/water partition coefficients log*P*.

Compound	log*P*
**1**	0.241
**2**	0.245
**3**	0.019
**4**	0.084
**5**	0.251

## Data Availability

The original contributions presented in this study are included in the article/Supplementary Materials. Further inquiries can be directed to the corresponding author(s).

## Supplementary Materials

The following supporting information can be downloaded at: https://www.mdpi.com/article/10.3390/molecules31132336/s1.
