# Supplementary material for: Asymmetrically Disubstituted Pyrenebutyrate Complexes of Pt(IV) as Cisplatin Prodrugs with Improved Anticancer Activity
Source: Molecules. 2026 Jul 3;31(13):2336. doi: 10.3390/molecules31132336 (PMC13362905; doi:10.3390/molecules31132336)
Supplement: Supplementary file 1 [file molecules-31-02336-s001.zip › molecules-4385143-supplementary.pdf]

Supplementary materials to

Asymmetrically disubstituted pyrenebutyrate complexes of Pt(IV) as cisplatin prodrugs with improved anticancer activity

Rositsa Mihaylova [1], Veronika Mihaylova [2], Nikola Burdzhiev[2], Ivo D. Ivanov [2], Zhanina Petkova [3], Georgi Momekov [1] Denitsa Momekova [1] and Anife Ahmedova[2,*]

[1] Faculty of Pharmacy, Medical University - Sofia, 2 Dunav Street, 1000 Sofia, Bulgaria; rmihaylova@pharmfac.mu-sofia.bg, gmomekov@pharmfac.mu-sofia.bg, dmomekova@pharmfac.mu-sofia.bg,

[2] Faculty of Chemistry and Pharmacy Sofia University, 1, J. Bourchier blvd., Sofia 1164, Bulgaria; Ahmedova@chem.uni-sofia.bg; ahvm@chem.uni-sofia.bg; ohnb@chem.uni-sofia.bg, ahidi@chem.uni-sofia.bg;

[3] Institute of Organic Chemistry with Centre of Phytochemistry, Bulgarian Academy of Sciences, Acad. G. Bonchev str. Bl. 9, 1113 Sofia, Bulgaria, Zhanina.Petkova@orgchm.bas.bg

* Correspondence: Ahmedova@chem.uni-sofia.bg ; Tel.: +359 2-8161-247

Content

IR Spectra of all Pt(IV) complexes 2

HR-ESI-MS Spectra of the studied complexes **2** – **5** 5

NMR Spectra of the studied complexes **2** – **5** 9

Stability of the complexes in presence of the biological reductant, glucose or ascorbic acid, and followed by NMR, fluorescence or HPLC measurements 14

IR Spectra of all Pt(IV) complexes

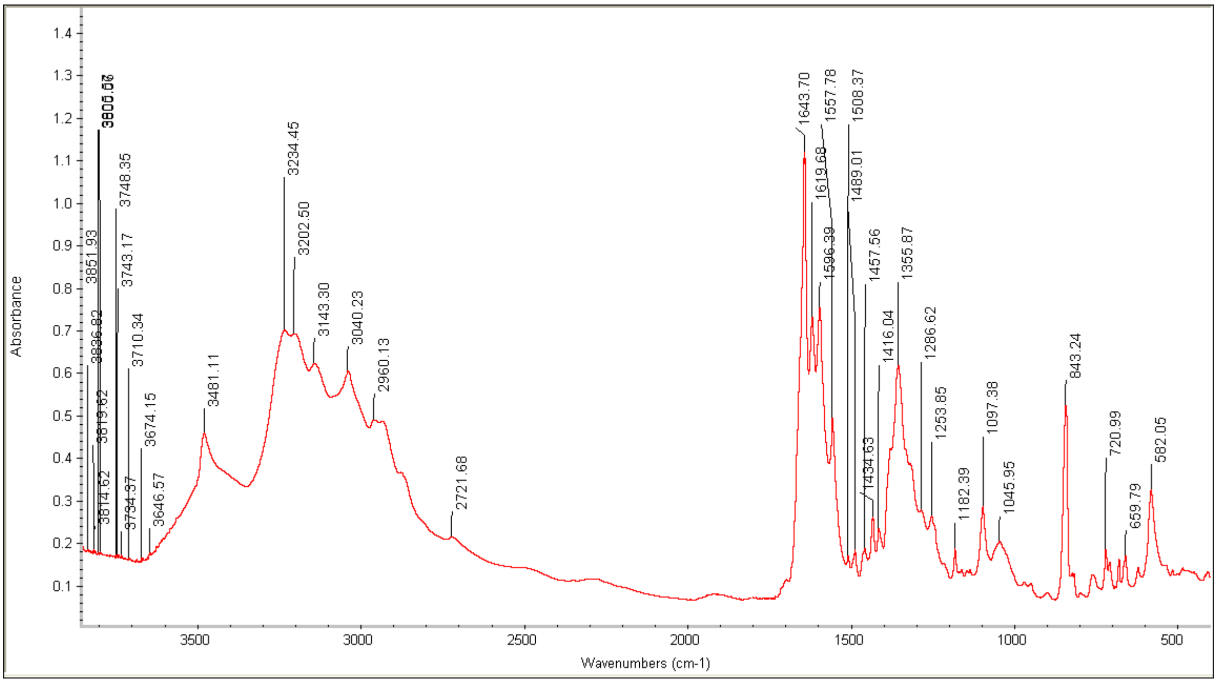

Figure S1. IR spectrum of complex **1** (AAt13).

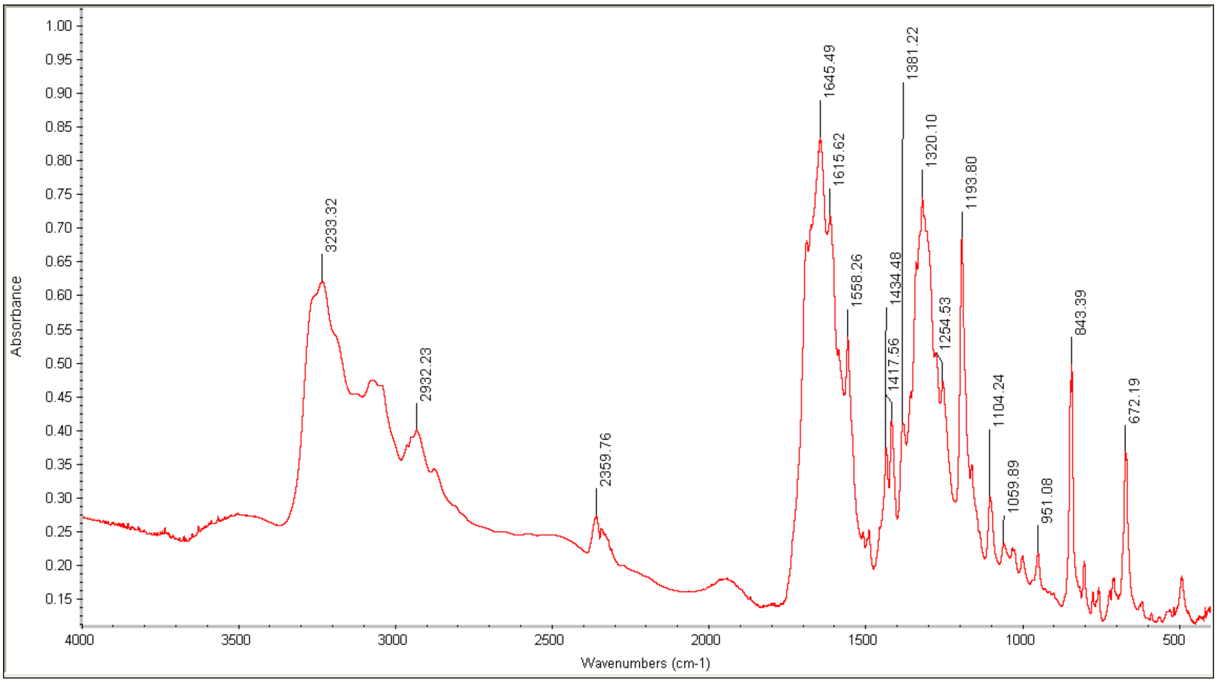

Figure S2. IR spectrum of complex **2** (AAt22).

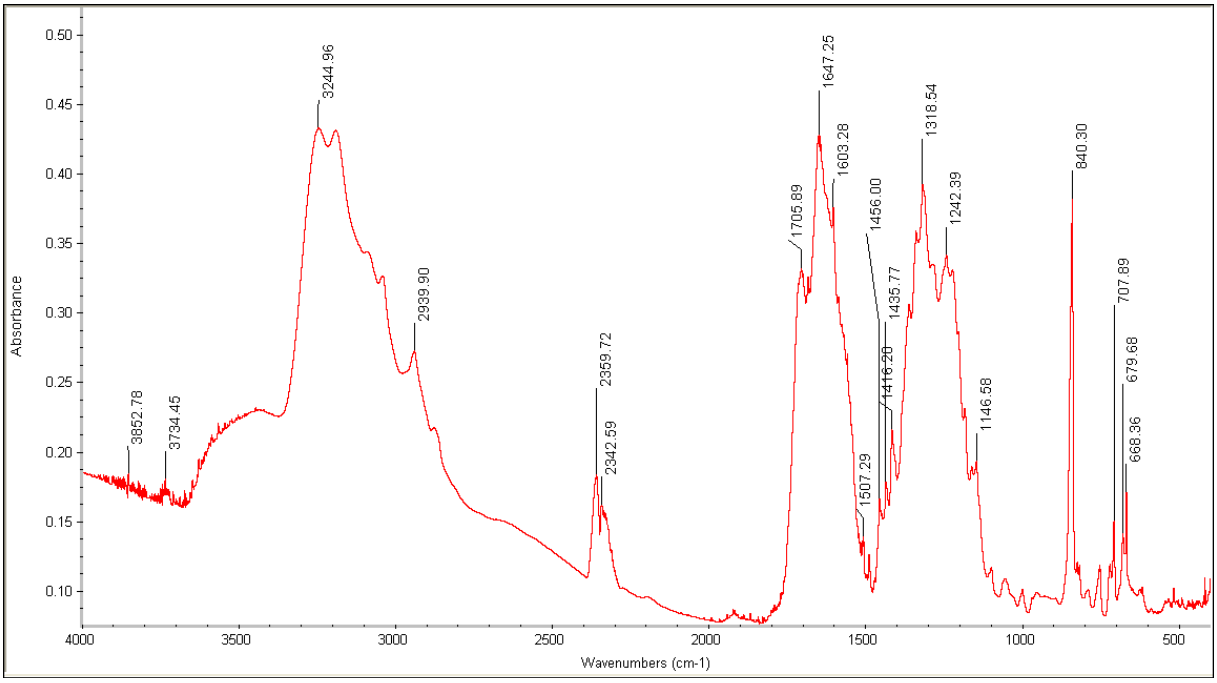

Figure S3. IR spectrum of complex **3** (AAt15).

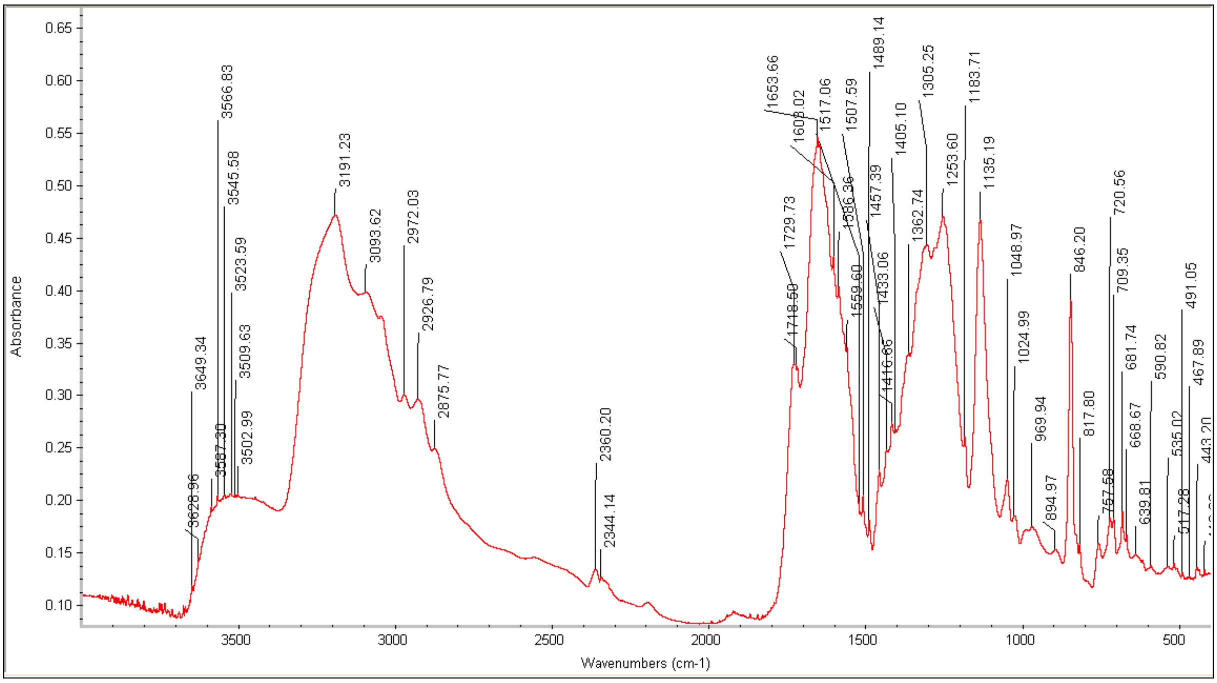

Figure S4. IR spectrum of complex **4** (AAt14).

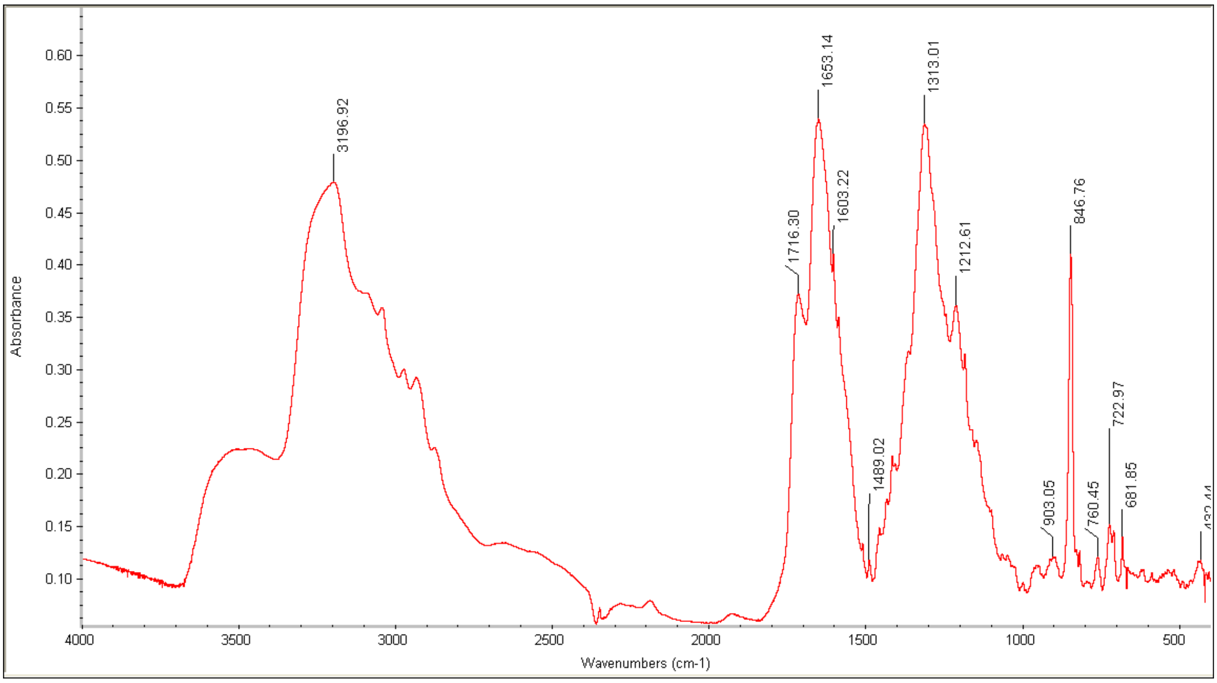

Figure S5. IR spectrum of complex **5** (AA33).

HR-ESI-MS Spectra of the studied complexes **2** – **5**

A

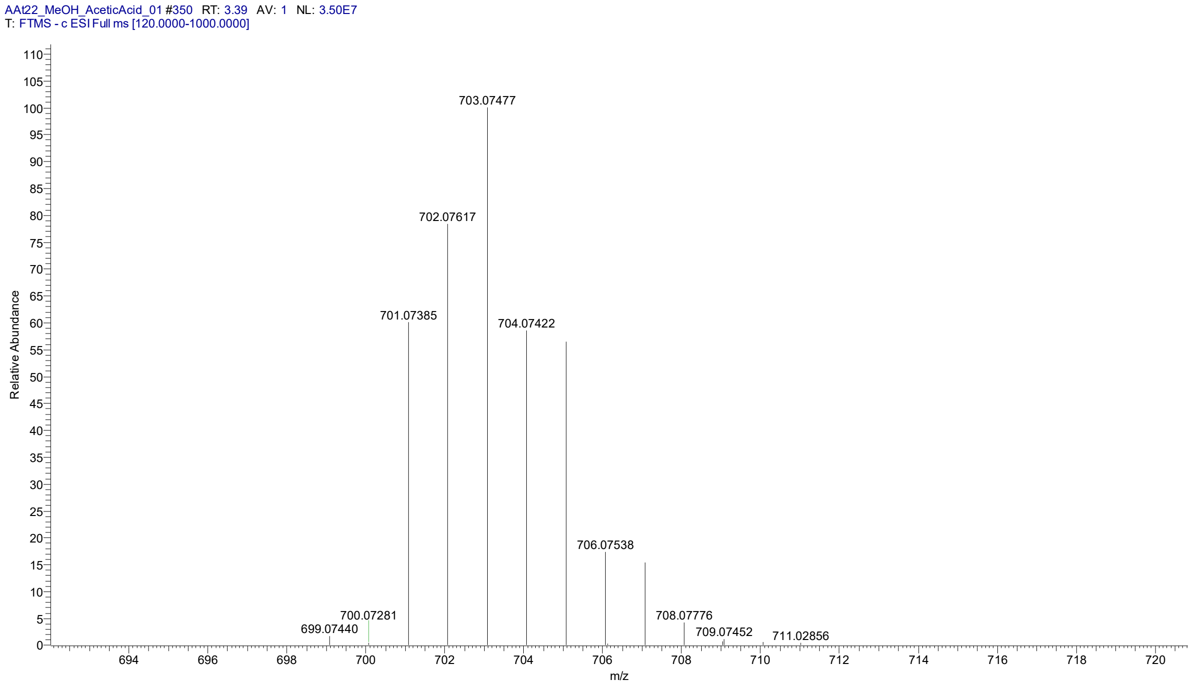

B

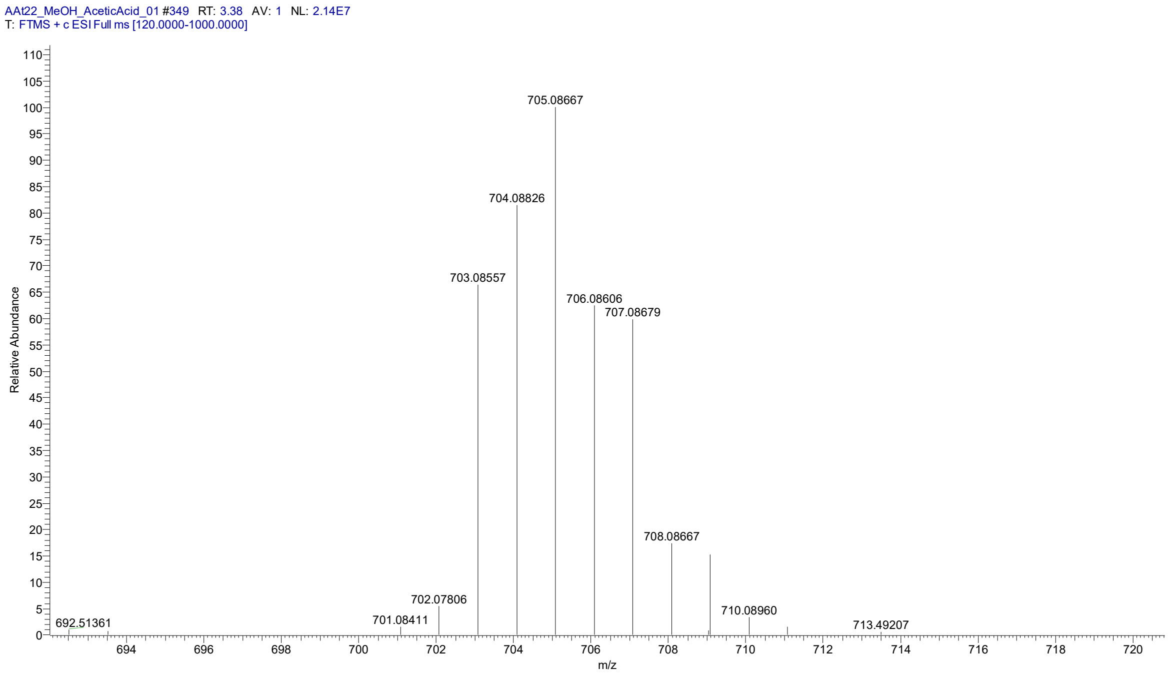

Figure S6. HR-ESI-MS spectrum of complex **2** (AAt22) : **A** (-) mode; **B** (+) mode.

A

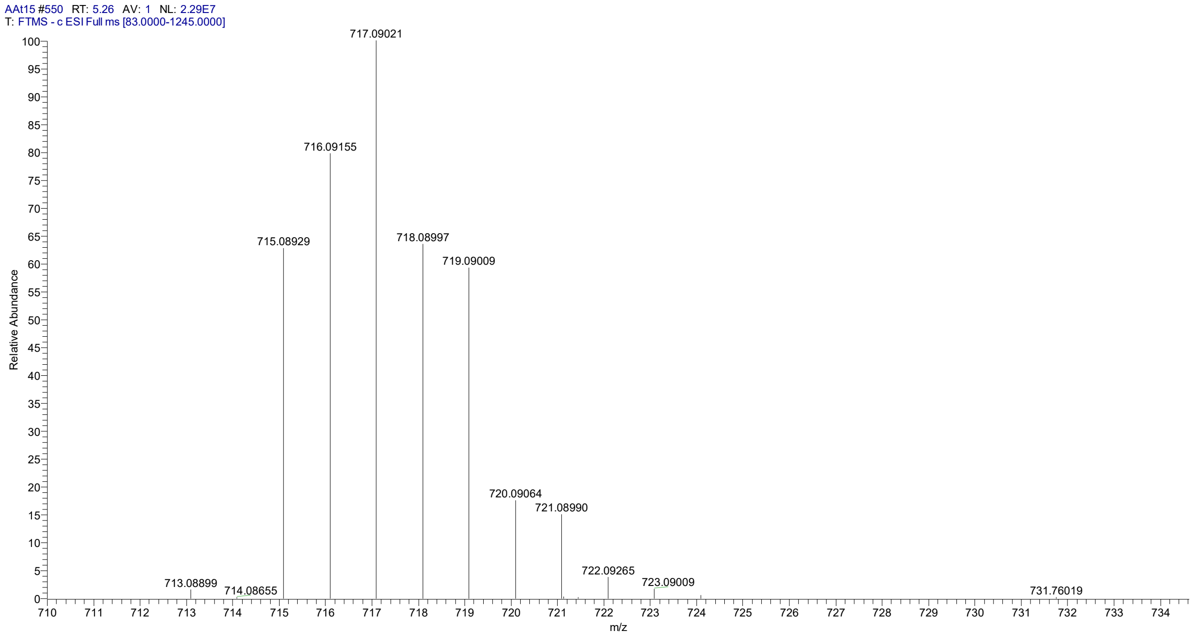

B

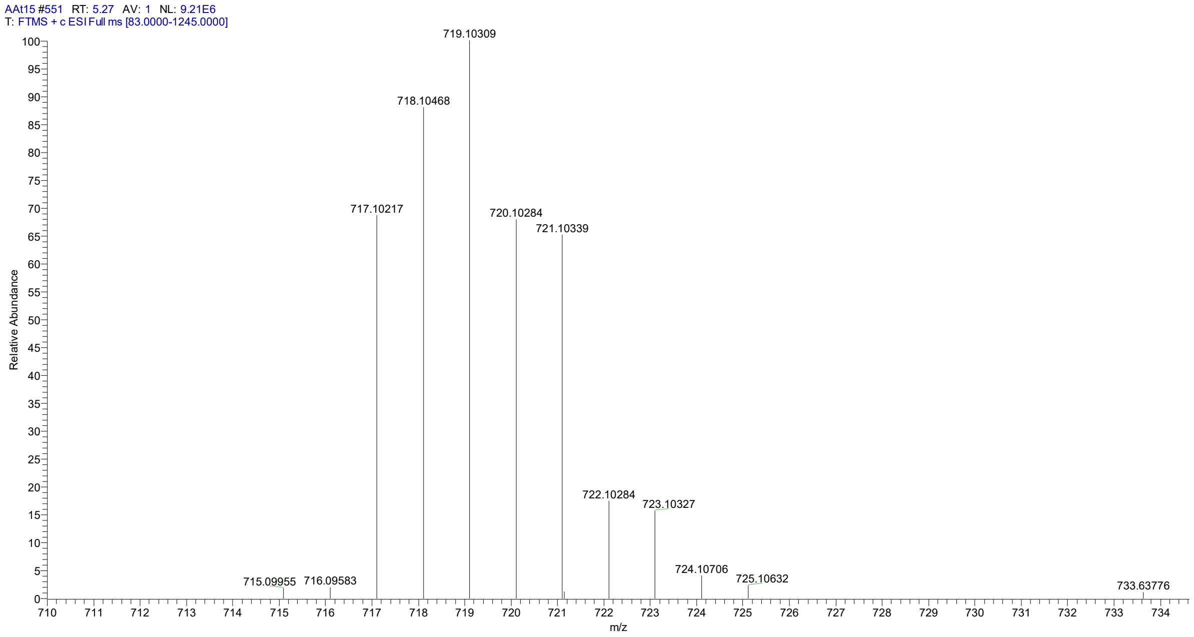

Figure S7. HR-ESI-MS spectrum of complex **3** (AAt15) : **A** (-) mode; **B** (+) mode.

A

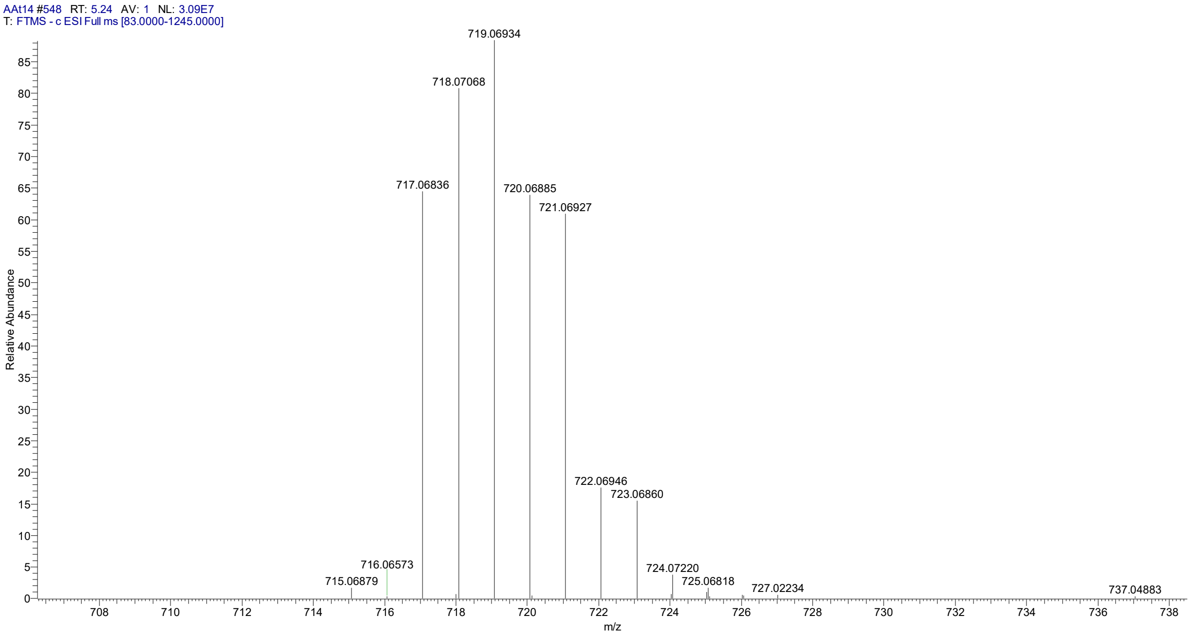

B

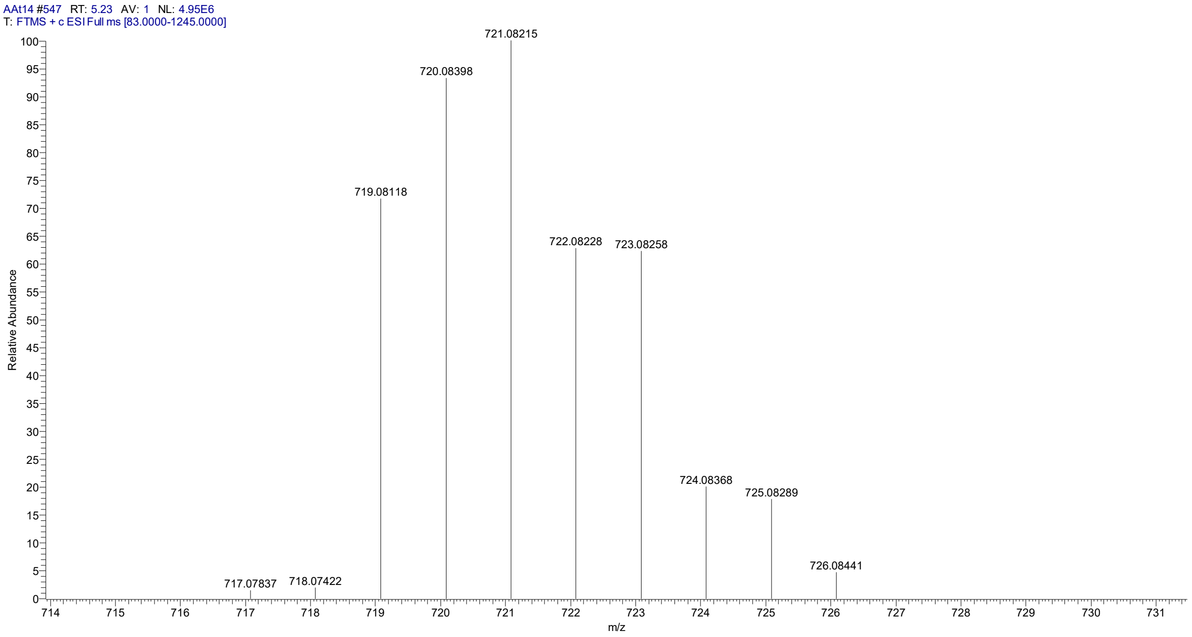

Figure S8. HR-ESI-MS spectrum of complex **4** (AAt14) : **A** (-) mode; **B** (+) mode.

A

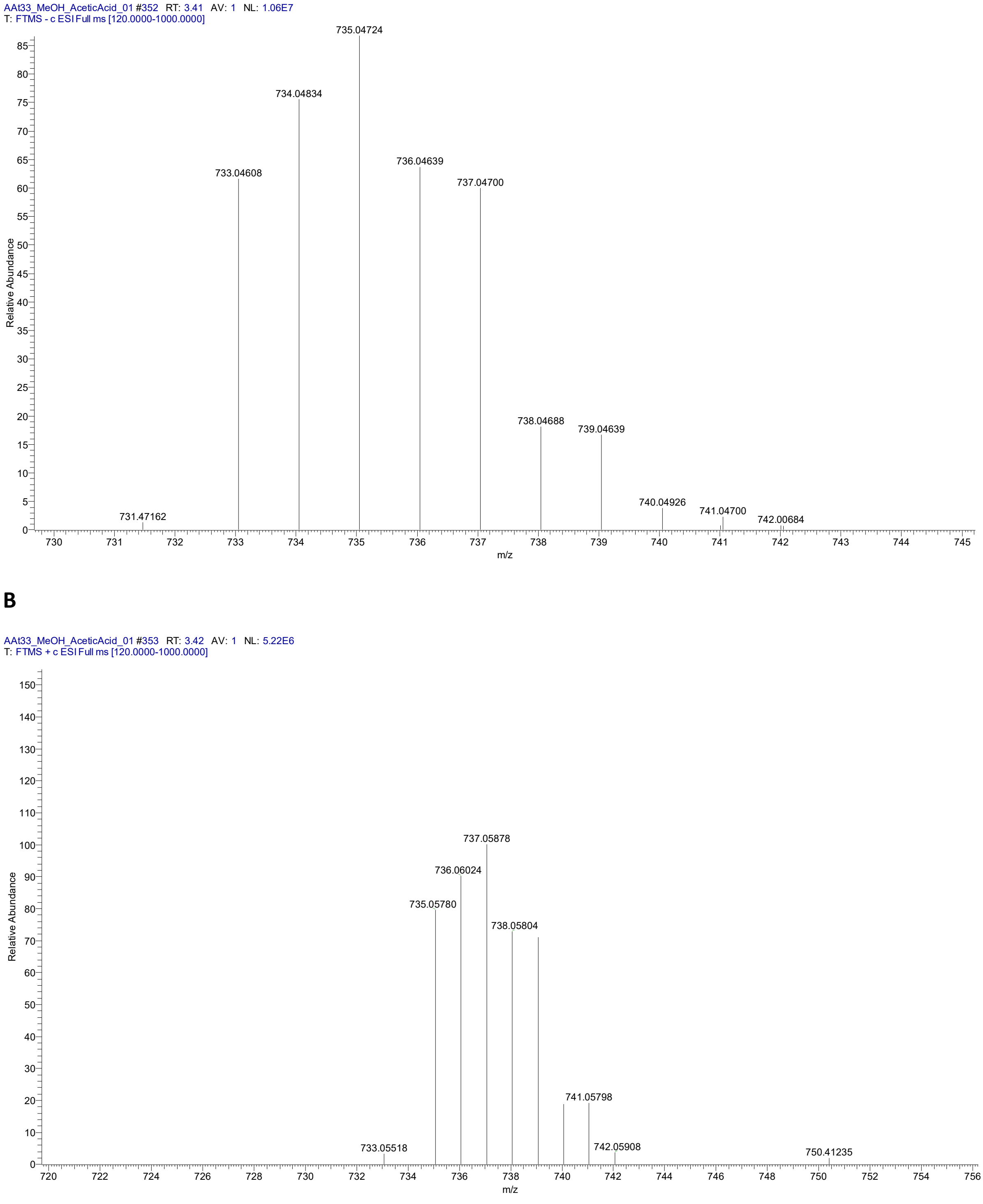

Figure S9. HR-ESI-MS spectrum of complex **5** (AA33) : **A** (-) mode; **B** (+) mode.

NMR Spectra of the studied complexes 2 – 5

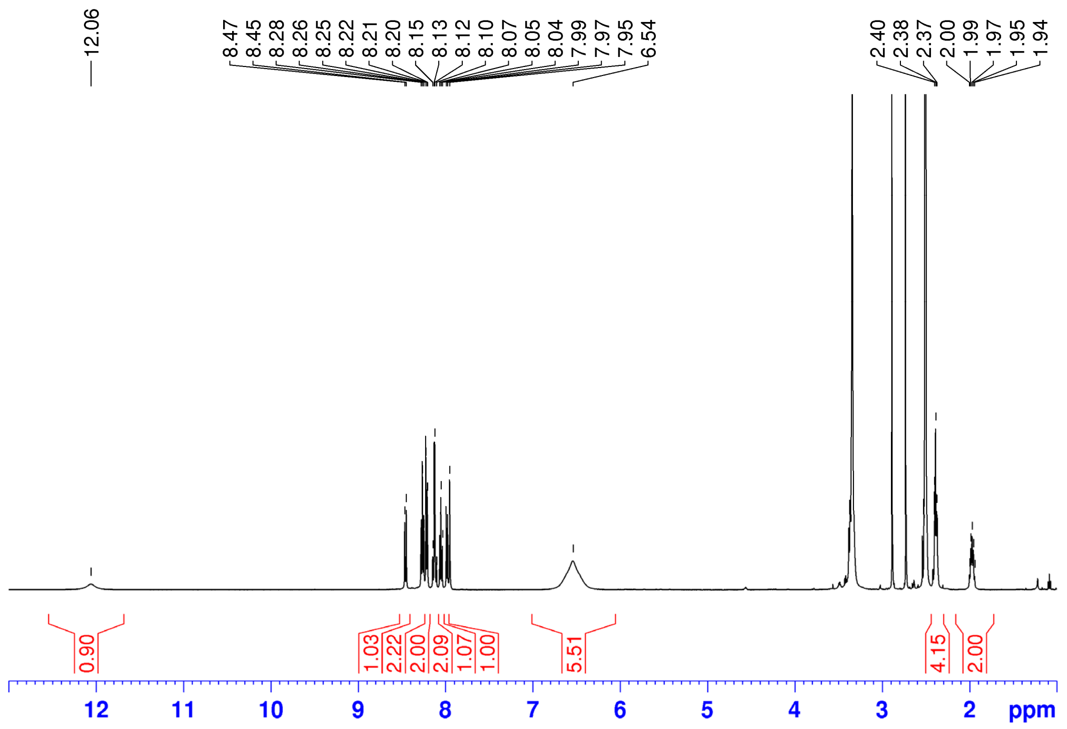

Figure S10. ^{1}H NMR spectrum of complex **2**.

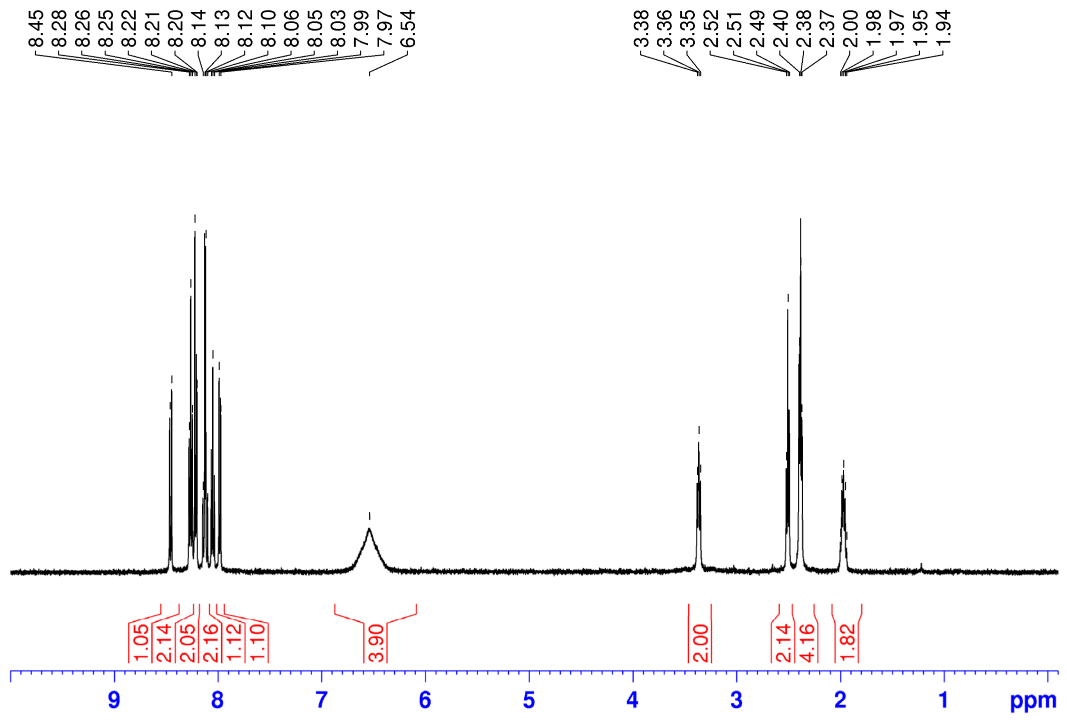

Figure S11. 1D DOSY NMR spectrum of complex **2**.

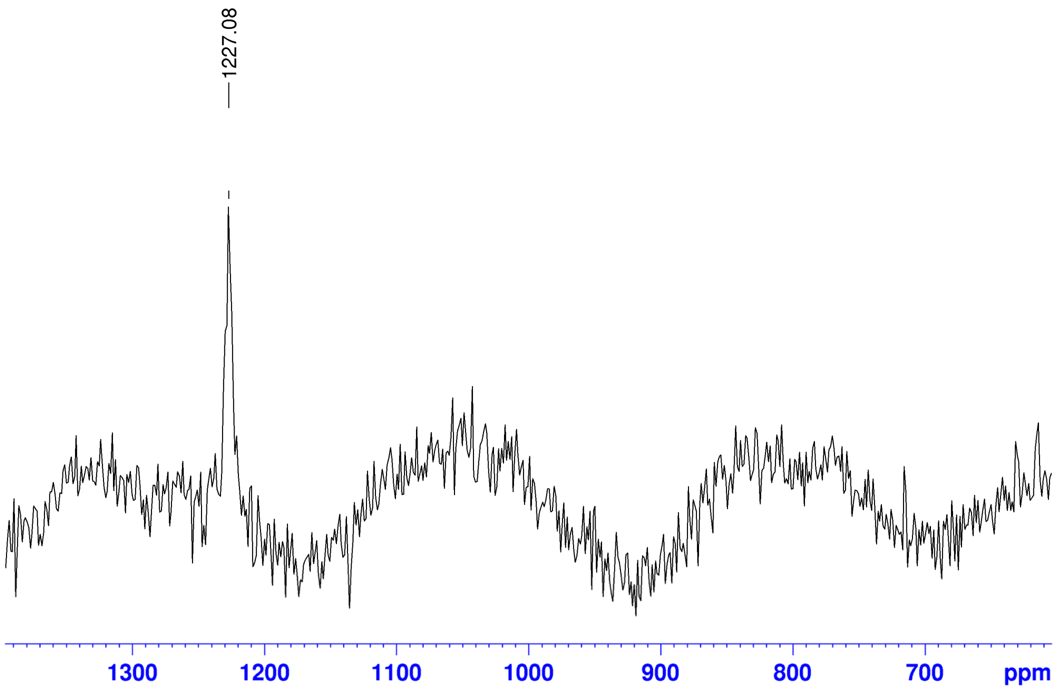

Figure S12. ^{195}Pt NMR spectrum of complex **2**.

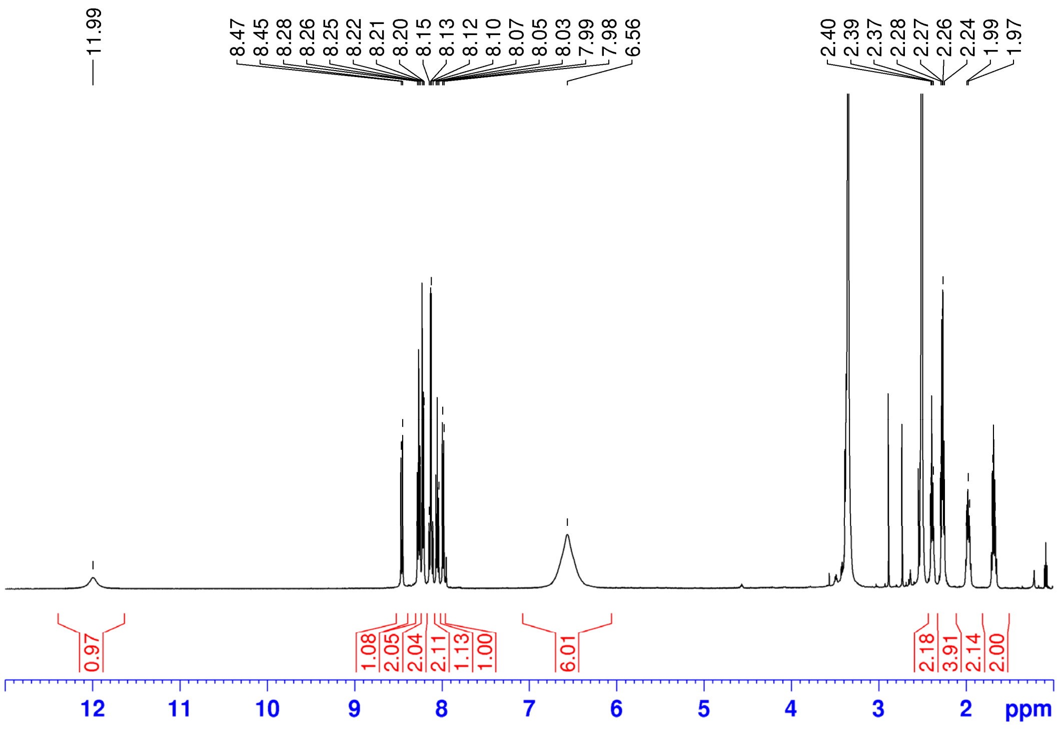

Figure S13. ^{1}H NMR of complex **3**.

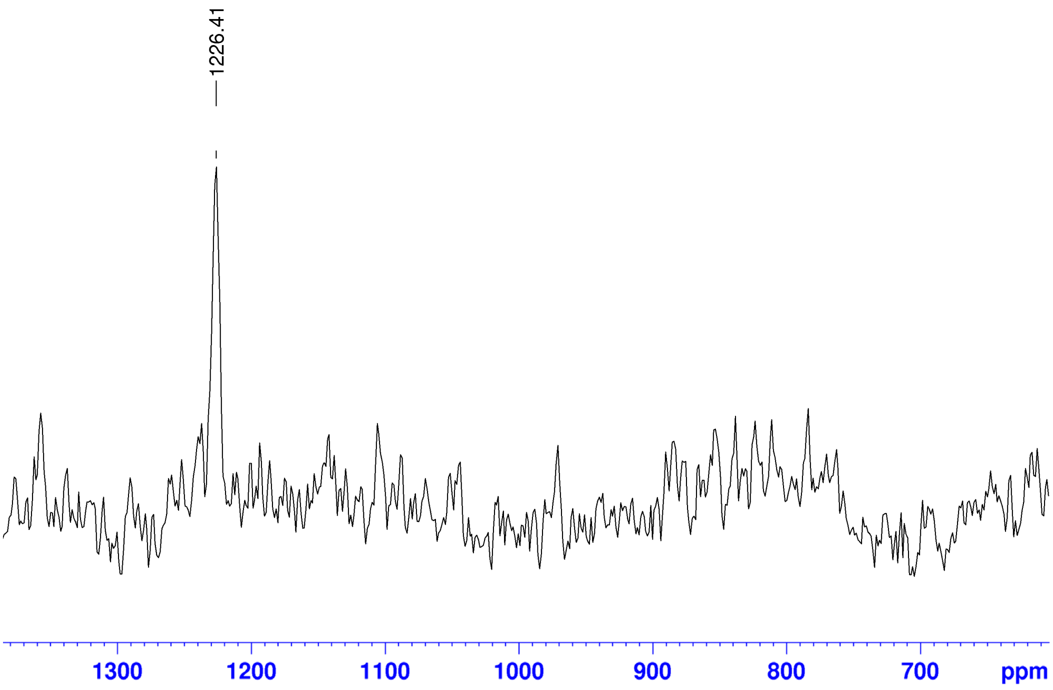

Figure S14. ^{195}Pt NMR of complex **3.**

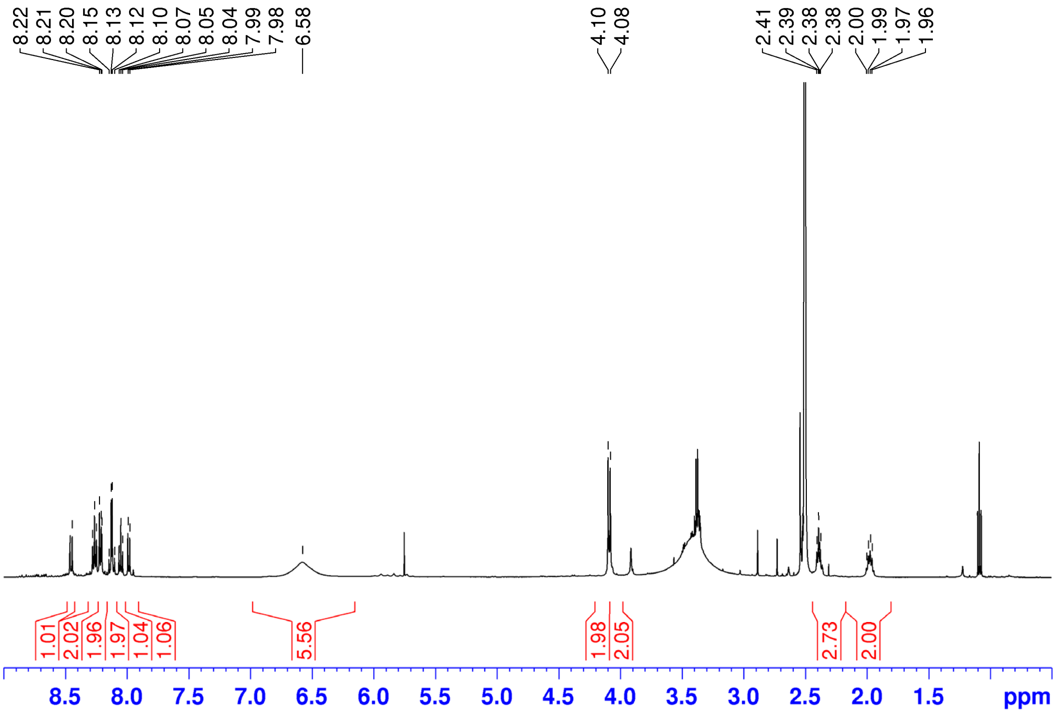

Figure S15. ^{1}H NMR of complex **4**.

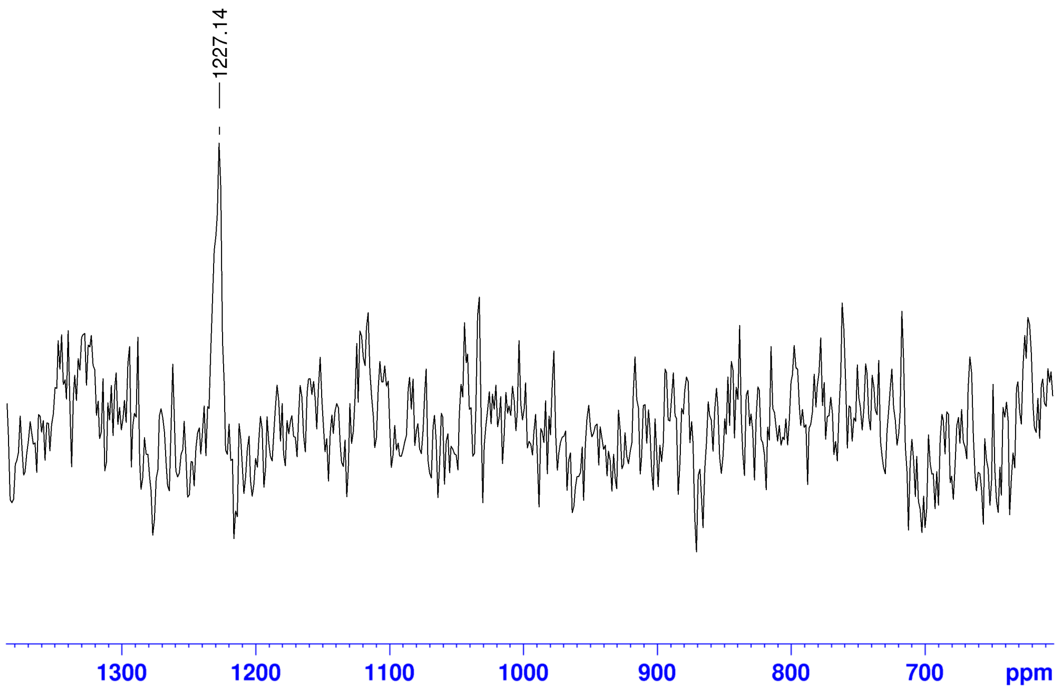

Figure S16. ^{195}Pt NMR of complex **4.**

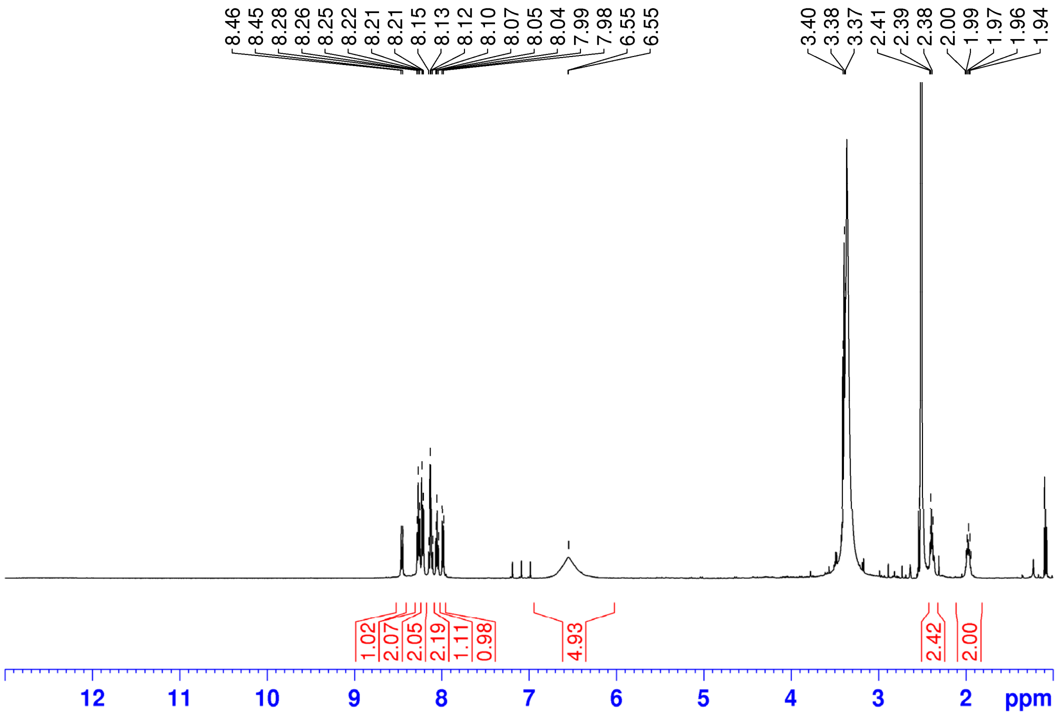

Figure S17. ^{1}H NMR of complex **5**.

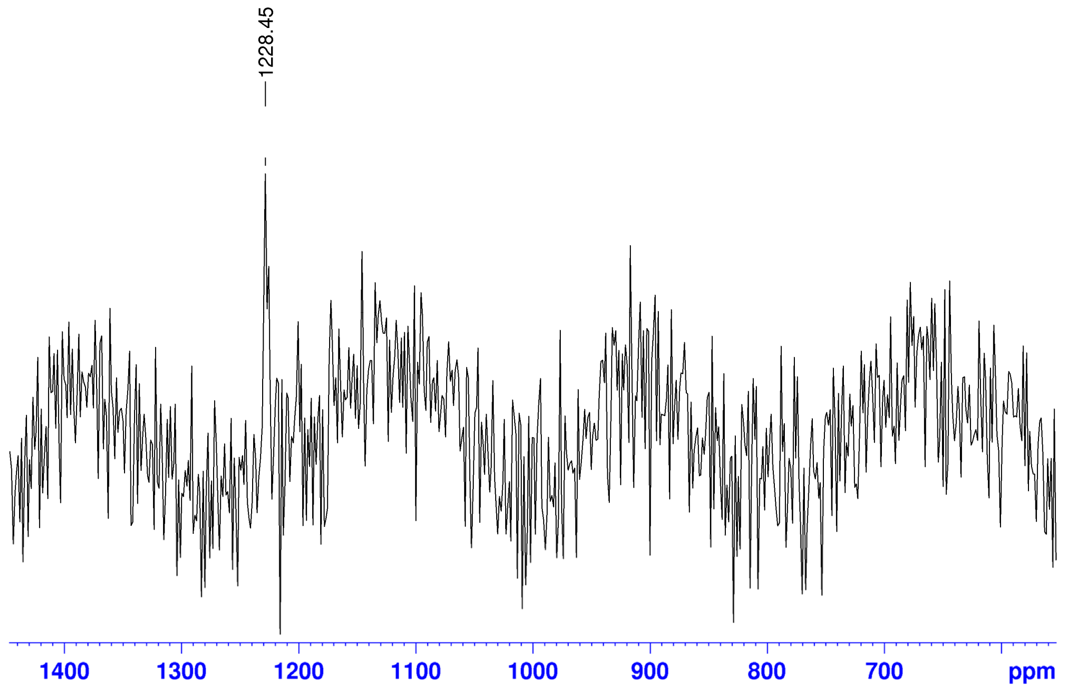

Figure S18. ^{195}Pt NMR of complex **5.**

Stability of the complexes in presence of the biological reductant, glucose or ascorbic acid, and followed by NMR, fluorescence or HPLC measurements

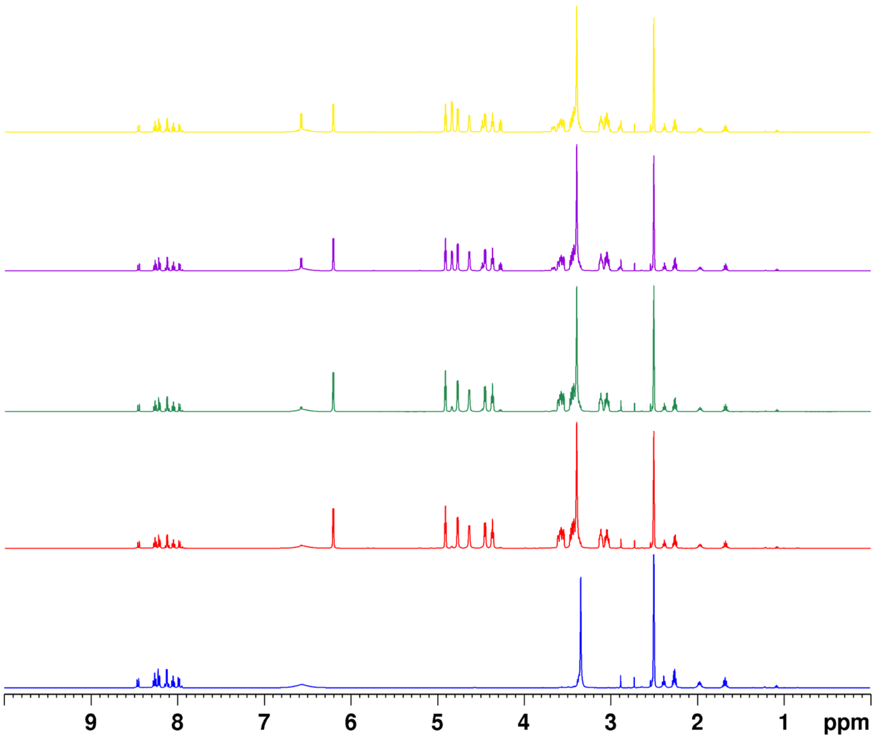

Figure S19. Monitoring of the ^{1}H-NMR spectra for over 92 hours after addition of glucose to complex **3** (in DMSO-d_6).

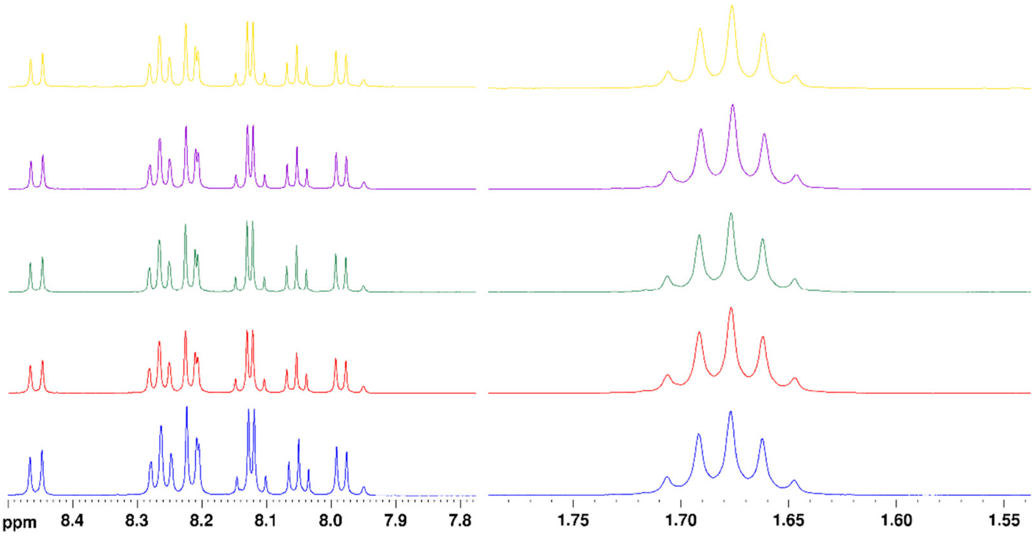

Figure S20. Monitoring of the ^{1}H-NMR spectra for over 92 hours after addition of glucose to complex **3** (in DMSO-d_6) is shown in two different regions – ca. 8.40 ppm (left) and ca. 2.02 ppm (right)

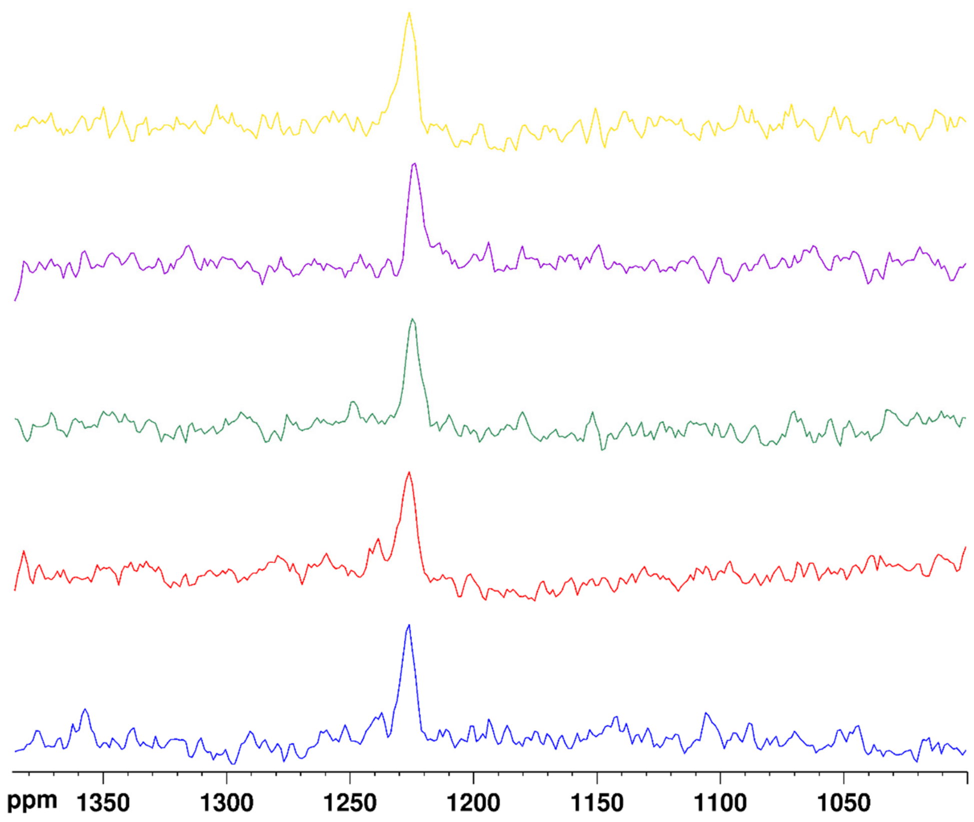

Figure S21. Monitoring of the ^{195}Pt-NMR spectra for over 92 hours after addition of glucose to complex **3** (in DMSO-d_6).

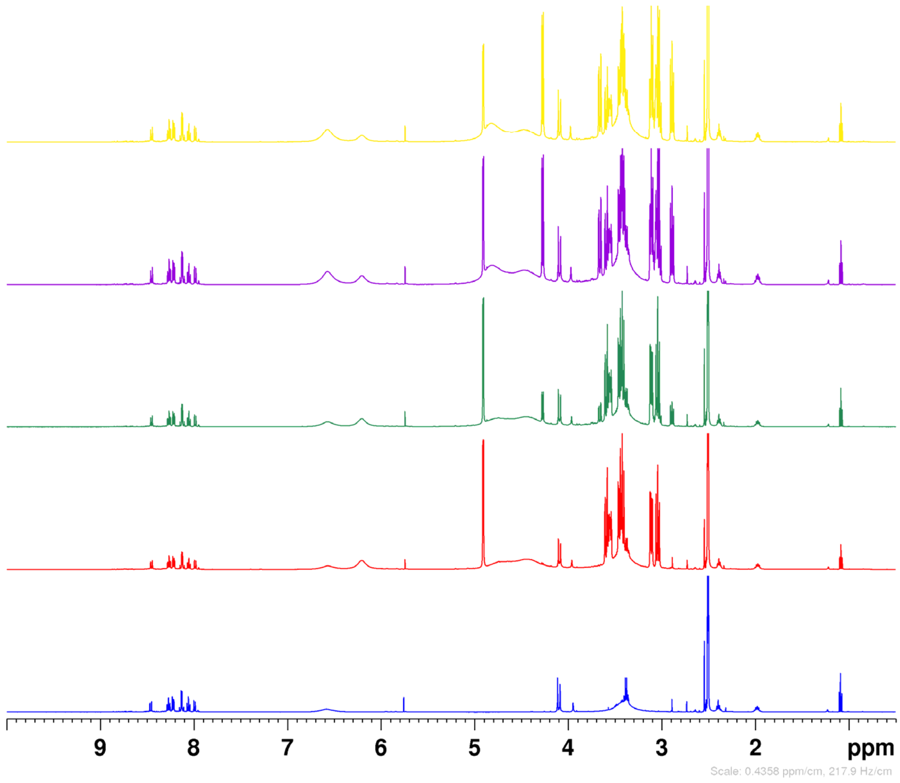

Figure S22. Monitoring of the ^{1}H-NMR spectra for over 92 hours after addition of glucose to complex **4** (in DMSO-d_6).

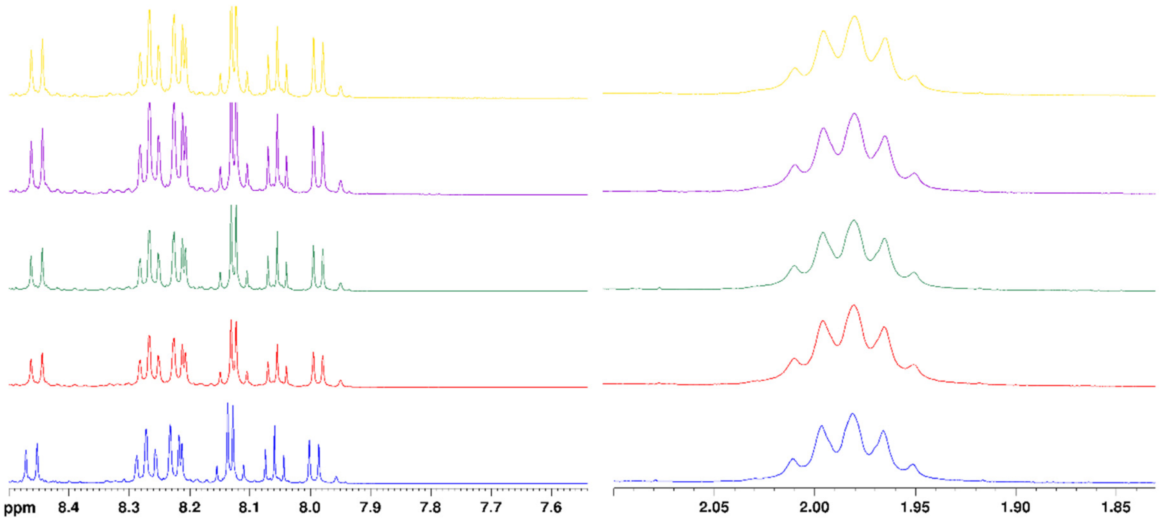

Figure S23. Monitoring of the ^{1}H-NMR spectra for over 92 hours after addition of glucose to complex **4** (in DMSO-*d*$_6$) shown in two different regions – ca. 8.40 ppm (left) and ca. 2.02 ppm (right) .

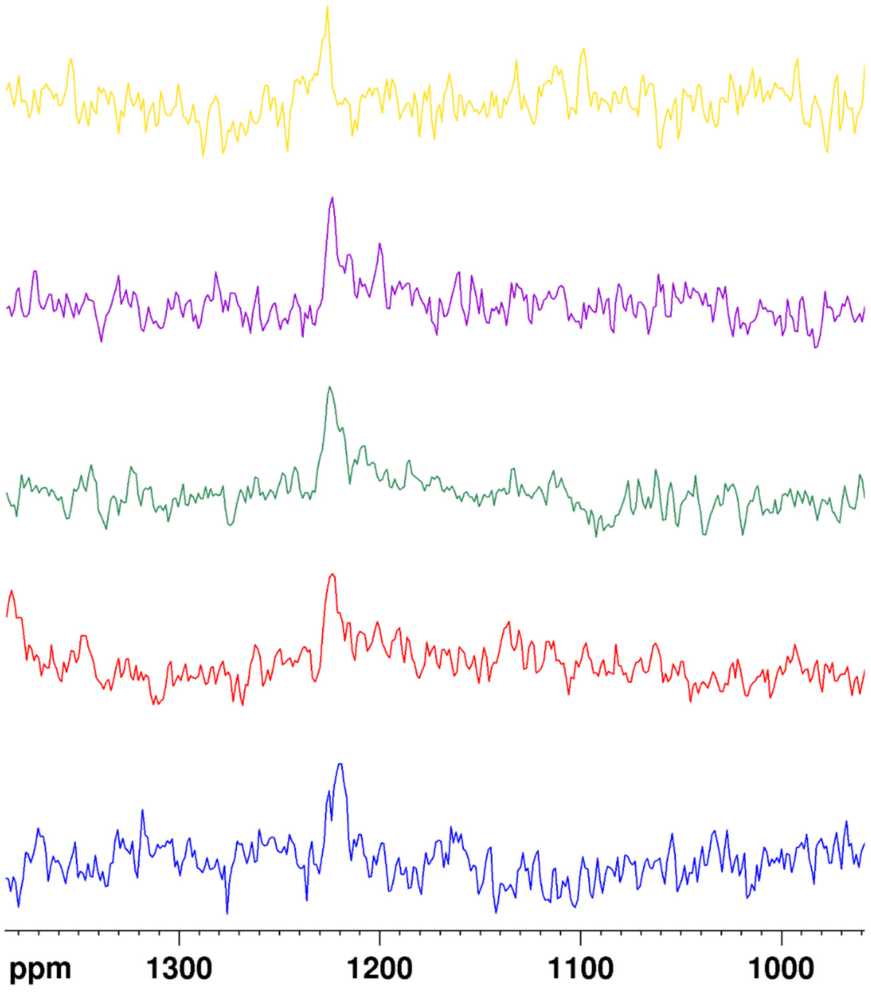

Figure S24. Monitoring of the ^{195}Pt-NMR spectra for over 92 hours after addition of glucose to complex **4** (in DMSO-*d*$_6$).

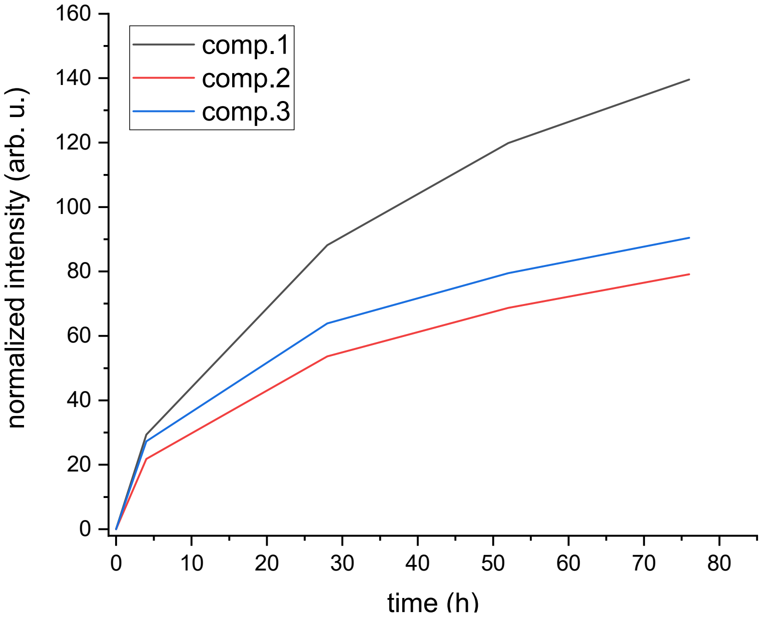

Figure S25. Reduction by ascorbic acid of complexes **1** – **3** (in water-to-DMSO ratio of 1:2) upon incubation in 10 fold excess for 3 days.

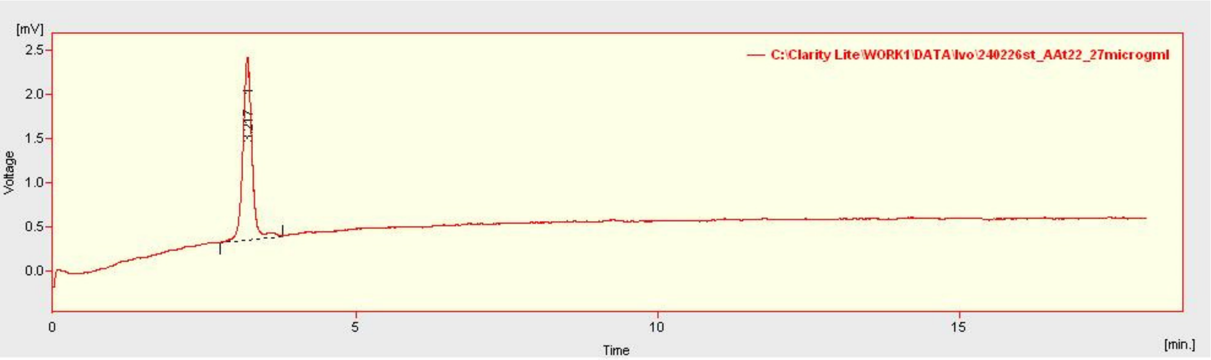

Figure S26. Representative chromatogram of complex **2.**

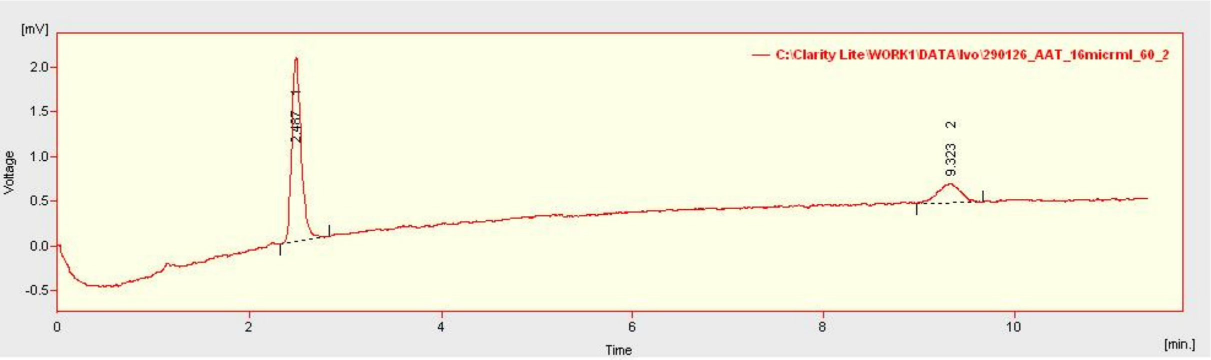

Figure S27. Representative chromatogram of the reduction of complex **3** with ascorbic acid.

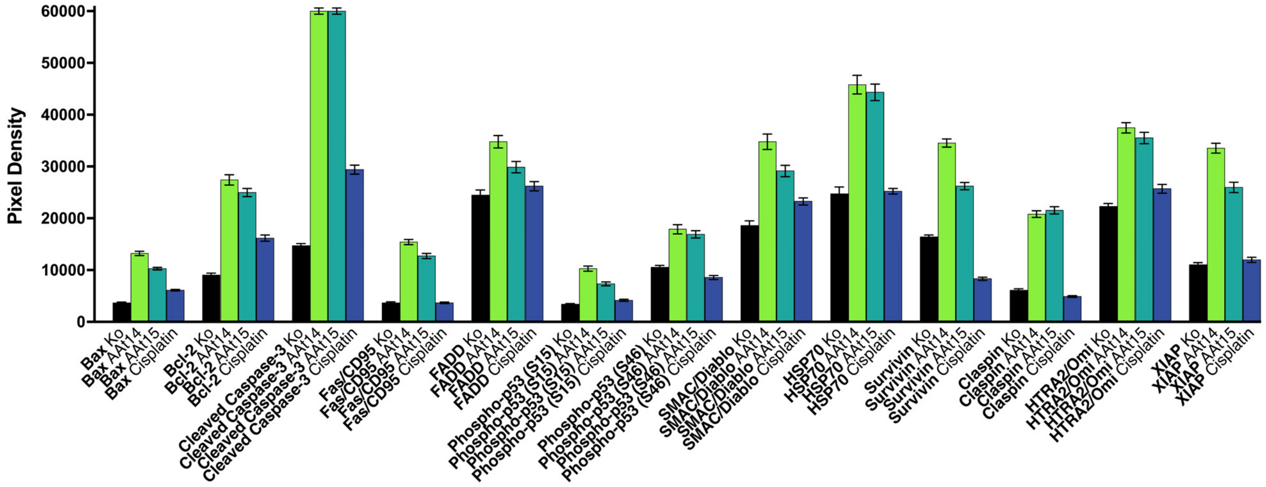

Figure S28. Changes in apoptosis-related protein expression in BV-173 cells following treatment with **4** (AAt14), **3** (AAt15) and cisplatin, compared to untreated naïve control (Ko). Bar graphs represent the mean pixel density values of the duplicate spots corresponding to each protein on the membrane array, determined by ImageJ densitometric analysis after 48 h exposure at IC_{50} concentrations. Statistical significance was evaluated using one-way ANOVA analysis relative to the untreated control (*$p < 0.05$).
